# Immunome differences between porcine ileal and jejunal Peyer’s patches revealed by global transcriptome sequencing of gut-associated lymphoid tissues

**DOI:** 10.1038/s41598-018-27019-7

**Published:** 2018-06-13

**Authors:** T. Maroilley, M. Berri, G. Lemonnier, D. Esquerré, C. Chevaleyre, S. Mélo, F. Meurens, J. L. Coville, J. J. Leplat, A. Rau, B. Bed’hom, S. Vincent-Naulleau, M. J. Mercat, Y. Billon, P. Lepage, C. Rogel-Gaillard, J. Estellé

**Affiliations:** 1 GABI, INRA, AgroParisTech, Université Paris-Saclay, 78350 Jouy-en-Josas, France; 2 ISP, INRA, Université de Tours, 37380 Nouzilly, France; 3 GenPhySE, INRA, INPT, ENVT, Université de Toulouse, 31326 Castenet-Tolosan, France; 4 LREG, IRCM, DRF, CEA, Université Paris-Saclay, 78350 Jouy-en-Josas, France; 5 BIOPORC and IFIP-Institut du porc, La Motte au Vicomte, BP 35104, 35651 Le Rheu, France; 6 GENESI, INRA, 17700 Surgères, France; 7 MICALIS Institute, INRA, AgroParisTech, Université Paris-Saclay, 78350 Jouy-en-Josas, France; 8 Present Address: BIOEPAR, INRA, Oniris, La Chantrerie, 44307 Nantes France

## Abstract

The epithelium of the intestinal mucosa and the gut-associated lymphoid tissues (GALT) constitute an essential physical and immunological barrier against pathogens. In order to study the specificities of the GALT transcriptome in pigs, we compared the transcriptome profiles of jejunal and ileal Peyer’s patches (PPs), mesenteric lymph nodes (MLNs) and peripheral blood (PB) of four male piglets by RNA-Seq. We identified 1,103 differentially expressed (DE) genes between ileal PPs (IPPs) and jejunal PPs (JPPs), and six times more DE genes between PPs and MLNs. The master regulator genes *FOXP3*, *GATA3*, *STAT4*, *TBX21* and *RORC* were less expressed in IPPs compared to JPPs, whereas the transcription factor *BCL6* was found more expressed in IPPs. In comparison between IPPs and JPPs, our analyses revealed predominant differential expression related to the differentiation of T cells into Th1, Th2, Th17 and iTreg in JPPs. Our results were consistent with previous reports regarding a higher T/B cells ratio in JPPs compared to IPPs. We found antisense transcription for respectively 24%, 22% and 14% of the transcripts detected in MLNs, PPs and PB, and significant positive correlations between PB and GALT transcriptomes. Allele-specific expression analyses revealed both shared and tissue-specific *cis*-genetic control of gene expression.

## Introduction

The digestive tract provides nutrients and energy from food to the organism, and it is also a major potential entry site for pathogens. The epithelium of the intestinal mucosa, together with its associated immune system, are in direct contact with both symbiotic and pathogenic microorganisms and thus constitute an essential immunological and physical barrier^1^. The specialized intestine immune system comprises the gut-associated lymphoid tissues (GALT) that include the Peyer’s patches (PPs), the mesenteric lymph nodes (MLNs) and isolated lymphoid follicles. PPs and MLNs both act as secondary lymphoid organs specifically associated with the gut tissues^2^. MLNs are induction sites of immune responses, as they initiate peptide presentation to lymphocytes by antigen presenting cells arriving from the intestine^2^. The PPs are distributed along the ileum and jejunum segments and they are thus classified as either ileal PPs (IPPs) or jejunal PPs (JPPs). The PPs are covered with the follicle-associated epithelium that harbors microfold cells (M cells). The sub-epithelial dome region contains abundant professional antigen presenting cells including dendritic cells (DCs) that take up antigens retrieved by M cells from the lumen of the digestive tract. The DCs present peptides to the T and B cells located in the PPs and trigger the initiation of antigen-specific immune responses and adaptive immunity. The PPs are also the primary inductive sites where most IgA immune responses are initiated by the class-switching of B cells from IgM to IgA antibodies^3^.

In humans, JPPs and IPPs are multiple isolated follicles, but in other species such as ruminants and pigs, JPPs are isolated follicles while IPPs are instead continuous structures along the ileum^4^. The overall anatomical differences between JPPs and IPPs in pigs suggest functional differences and their specific roles still need to be clarified^5,6^. Indeed, several studies have described histological and functional differences between the two porcine PPs. For instance, the cellular composition of the GALT has been described as follows^7^: B cells represent 90% and 45% of IPP and JPP cells, respectively, and 35% of MLN cells. Other studies have explored the functional and transcriptomic disparities for limited gene sets. For instance, it has been reported that IPPs would not be necessary for the development of the B cell systemic pool^8^ or specific IgA production^9^. In contrast, in a targeted gene expression approach, Levast *et al*.^10^ showed differential expression of 29 immune genes between IPPs and JPPs and observed a more diverse IgA repertoire in IPPs. Similarly, Gourbeyre *et al*.^11^ focused on pattern recognition receptors involved in innate immunity and observed differential expression for *TLR2*, *6*, *7*, *9*, *10* and *NLRP3* between IPPs and JPPs.

The existing published results strongly suggest shared but subtle differences between the functionalities of IPPs and JPPs. To further explore this question, we are presenting in this work a study focusing on transcriptome profiles in pigs with no clinical signs of infection, by stranded RNA-sequencing of IPPs, JPPs, MLNs and peripheral blood as a complementary non-GALT immune tissue. We report the differential gene expression between PPs and MLNs, and more specifically between IPPs and JPPs, together with the analysis of antisense transcription and allele-specific expression (ASE) in the four tissues.

## Materials and Methods

### Animals and sample collection

All animals were Large White pigs bred in the INRA experimental farm at Le Magneraud (GENESI, UE 1372, France). All animals were weaned at 28 days of age and fed *ad libitum*. They belong to the previously reported SUS_FLORA cohort^12^ for which all animals were sampled for blood at 60 days of age. Among this cohort, 36 pigs were slaughtered at 70 days of age to sample IPPs, JPPs and MLNs. A subset of four uncastrated males was selected for the transcriptome study. The remaining group of 32 pigs that comprised 16 males and 16 females was used as a validation group for quantitative real-time PCR (qRT-PCR) studies. All animal experiments were carried out in accordance with European Guidelines for the Care and Use of Animals for Research Purposes. The animal protocol was approved by the local ethics committee in Poitou Charentes and assigned the approval number CE2013-2.

Peripheral blood (PB) was sampled in the jugular vein using PAXgene Blood RNA tubes (PreAnalytiX, Qiagen, Germany). Pieces of MLNs, IPPs and JPPs were collected, snap frozen in liquid nitrogen, and stored at −80 °C until use.

### RNA extraction and sequencing

Tissue RNA extractions were performed as previously reported^11,12^. In brief, 60 mg of tissue were lysed in 1 ml of Trizol (Invitrogen, Cergy Pontoise, France) with ceramic beads (Bertin technologies, St Quentin en Yvelines, France), and total RNA was purified using RNeasy Mini Kit (Qiagen, Courtaboeuf, France) according to the manufacturer’s recommendations. Residual genomic DNA was removed using DNase digestion with RNase-free DNase I Amplification Grade (Invitrogen, Cergy Pontoise, France) following the recommended protocol. For blood samples, no globin depletion procedure was applied, and RNA purification was performed as reported in Maroilley *et al*.^13^. One µg of total RNA was reverse transcribed for 90 min at 37 °C in a 20 µl volume containing 0.25 mM dNTP of OligodT, 25 U of MuMLV reverse transcriptase in 4 μl 5X MuMLV buffer (Eurogentec, Liège, Belgium). After heat-inactivation at 93 °C for 5 min, generated cDNA was stored at −80 °C until use. Finally, reverse-transcribed RNA samples of MLNs, IPPs, JPPs and PB from the four pigs chosen for transcriptome analysis were sequenced using three lanes in an Illumina HiSeq 2000 with the Illumina TruSeq Stranded mRNA Library Prep Kit. On average, 47 million reads (100 bp paired-end) were obtained per sample. The raw reads are available at NCBI’s SRA repository (Bioproject PRLNA416857; accessions SAMN07966348 to SAMN07966363).

### Differential expression analyses of RNA-Seq data

After quality control using the FastQC tool, sequencing reads were filtered with the Trimmomatic tool (v.0.32)^14^ by trimming leading and trailing bases with phred qualities less than 3 and dropping reads shorter than 36 bases long and those with average phred qualities per base less than 15. Reads were subsequently aligned on the reference genome Sscrofa10.2 using TopHat2 (v2.0.14), with the default parameters and the gene annotation available for *Sus scrofa* in the Ensembl 85 release database^15^. Based on the read mapping with TopHat2, gene expression was quantified by obtaining read counts with the HTSeq-count software (v.0.6.1p1)^16^ with a default parameter that discards all reads that map to multiple locations. Expression levels of antisense transcripts were also analyzed by using the strand read information. We quantified antisense transcription according to the number of reads mapping to the corresponding gene reference sequence on the opposite strand. Therefore, we did not use the annotation of antisense transcripts available in Ensembl 89 but only the annotation of reference genes with their genomic position. Read counts were further analyzed using the edgeR R/Bioconductor package (v.3.12.1)^17^. We retained genes as expressed in a tissue when the count per million (CPM) sense reads was greater than one for at least two animals. Similarly, a gene was found to have an antisense transcription if the antisense read CPM was greater than one for at least two animals. Read counts were then normalized according to the total number of reads of each sample using the Trimmed Mean of M-values normalization method (TMM)^18^ implemented in edgeR (Supplementary Tables S1 and S2). The sense and antisense expression data structures were explored with multi-dimensional scaling (MDS) plots. The correlation between sense and antisense transcription across tissues was estimated by Spearman correlation using the normalized read counts.

The differentially expressed (DE) genes between GALT tissues were detected by fitting a negative binomial generalized linear model (GLM) in edgeR. In order to take into account intra-individual variability, the model included covariates for both tissue and individual. A likelihood ratio test was performed to identify DE genes among each pair of tissues, and *P-values* were corrected for multiple testing using the Benjamini-Hochberg control of the False Discovery Rate (FDR < 0.05). Finally, smear plots of log-fold expression changes versus log-concentration were produced using the “plotSmear” function in the edgeR package.

### Joint genotyping and inference of allelic specific expression

For ASE analysis, we realigned reads along the reference genome Sscrofa10.2 using the STAR 2-pass protocol (v.2.4.0i)^19^. After marking duplicates, we split the reads, realigned them on indels and recalibrated the sequence data following the GATK (v3.7) Best Practices for calling variants in RNA-Seq data^20^, and as described in Maroilley *et al*.^13^. SNPs were called with the HaplotypeCaller of the GATK tool (v3.7) using all samples and setting a base quality score higher than 10 and a coverage greater than 10 reads. We then removed SNPs with a low minor allele count (less than 3 reads that harbor the alternative allele) using VCFtools (v0.1.12a)^21^. In order to limit alignment biases of reads due to the presence of alternative SNP alleles, we created a masked genome reference sequence with an N at each SNP position using the Bedtools maskfasta tool (v2-2.24.0) and re-aligned reads on the masked genome with the same STAR 2-pass protocol. Using the dataset from this second alignment, counts of reads covering each allele at selected SNPs were obtained using ASEReadCounter of GATK v3.7 with parameters ensuring an adequate coverage and quality (read depth greater than 10, mapping quality greater than 10, base quality greater than 2). We then adapted ASEReadCounter output for the format required by QuASAR v.0.1^22^ and used the associated statistical method to genotype individuals from multiple RNA-Seq samples (fitAseNullMulti) and determine for each animal the probability of each possible genotype according to the alleles found in each tissue (with a minimum read coverage equal to five). Finally, we performed binomial tests on allelic read counts to detect potential allelic imbalance for the SNPs found to be heterozygous in each pig. The calculated *P-values* were corrected for multiple testing using the Benjamini-Hochberg control of the False Discovery Rate (FDR < 0.05).

### Functional enrichment analysis

The *Sus scrofa* Ensembl gene IDs (release 89 corresponding to Sscrofa10.2 assembly) annotated from DE and ASE analyses were submitted to Biomart in order to identify lists of human orthologous genes for subsequent functional enrichment analyses. The annotation was further updated using the Ensembl 91 release (Sscrofa11.1 assembly). For 869 genes still lacking GeneName HUGO Genome Nomenclature Committee (HGNC) annotation, we aligned the protein sequences corresponding to the pig gene Ensembl ID against the human RefSeq protein database using BLASTP^23^. The GOrilla tool^24^ was used for enrichment analyses of expressed genes in each tissue. For DE genes, we used the Ingenuity Pathway Analysis tool (IPA; http://www/ingenuity.com) and the Kyoto Encyclopedia of Genes and Genomes (KEGG) pathway mapper^25^. SNPs and ASE SNPs were annotated with the Variant Effect Predictor tool (Ensembl).

### Quantitative real-time PCR assays

In order to validate the differential expression of a set of immune response mediator genes, qRT-PCRs were carried out on IPPs, JPPs and MLNs from a group of 70-day-old pigs. Three genes, *β-2-microglobulin* (*B2M*), *hydroxymethylbilane synthase* (*HMBS*) and *glyceraldehyde-3-phosphate dehydrogenase* (*GAPDH*), were used as reference genes because of their stable expression between samples as evaluated using the geNorm algorithm^26^. All gene primers (Supplementary Table S3) were designed using the Clone Manager 9 software (Scientific & Educational Software, Cary, NC, USA). For cDNA synthesis, one µg of total RNA was reverse transcribed for 90 min at 37 °C in a 20 µl volume containing 0.25 mM dNTP of OligodT, 25 U of MuMLV reverse transcriptase in 4 µl 5X MuMLV buffer (Eurogentec, Liège, Belgium). After heat-inactivation at 93 °C for 5 min, generated cDNA was stored at −80 °C until use. qRT-PCRs were performed on a Bio-Rad Chromo 4 apparatus (Bio-Rad, Hercules, CA, USA) using the Mesa Green qPCR MasterMix Plus for SYBR assay (Eurogentec, Liège, Belgium) as previously described^27–29^. All qRT-PCRs displayed efficiency between 90 and 110%, and expression data were given as relative values using modified delta delta Ct method^30^ after Genex macroanalysis (Bio-Rad, Hercules, CA, USA)^26^.

### Pyrosequencing validation of selected ASE signals

A subset of ASE SNPs annotated to six genes were selected for validation studies by pyrosequencing with a PyroMark Q24 instrument (Qiagen). Supplementary Table S4 shows primer sequences used for PCR amplification and subsequent sequencing. For each cDNA sample, PCRs were performed using the HotStart Taq polymerase following the conditions specified in Supplementary Table S4. The allele quantification was subsequently performed in the PyroMark Q24 system according to manufacturer’s instructions.

## Results

### Global transcription in GALT and peripheral blood

**Figure 1:**
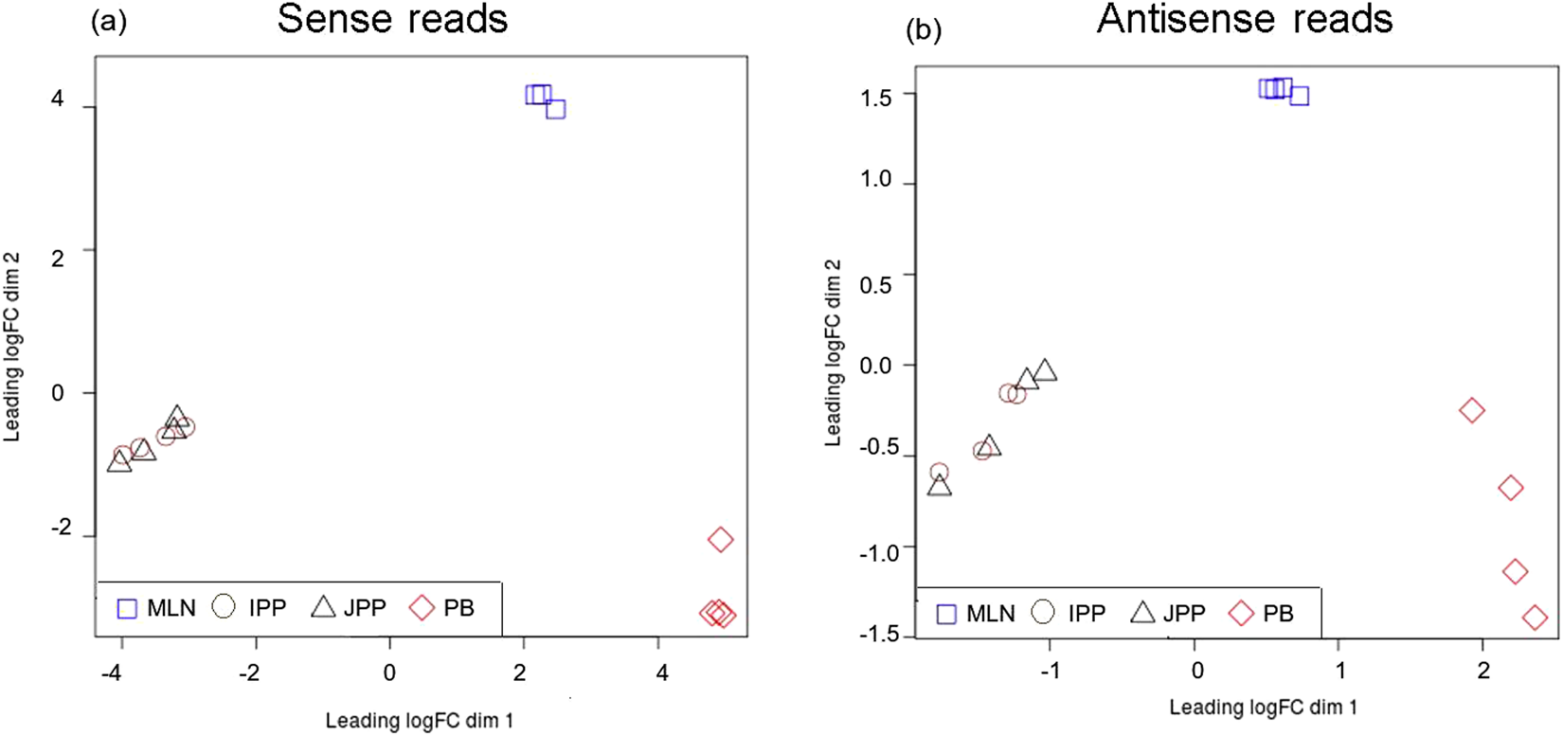
Summary plots of global gene expression analyses performed by RNA-Seq on MLN, IPP, JPP and PB. Graphs show MDS plots for normalized gene expression levels (**a**) and normalized antisense transcription levels (**b**).

The transcriptome analyses were carried out by RNA-Seq on the same four male piglets for blood samples (60-day-old), and for MLNs, IPPs and JPPs (70-day-old). The global expression data structure was explored with a multi-dimensional scaling plot (MDS plot, Fig. 1a) based on the TMM normalized gene expressions for each sample. In this two-dimensional plot, samples are positioned according to the statistical distance of their expression profiles, highlighting here that samples are grouped rather by tissue than by individual. Similar results were obtained from normalized antisense read counts (Fig. 1b). For both sense and antisense transcription levels, we observed a clear separation between MLNs, PB and PPs, whereas IPPs and JPPs grouped together, thus revealing strong similarities between PP types at this scale of comparison.

Globally, we found over 13,000 expressed genes (according to the pig genome annotation) in IPPs, JPPs and MLNs. In PB, only ~9,200 genes were detected (Table 1), which is likely an under-estimation since no globin depletion procedure was applied on blood samples, thus limiting the detection of weakly expressed genes due to an over-representation of globin genes. Indeed, HTSeq-count counted over 18 million reads for the *HBB* (*ENSSSCG00000014725*) gene and almost 12 million reads for the *HBA* (*ENSSSCG00000007978*) gene, corresponding to 43% and 27% of the reads, respectively. Additionally, an *HBB*-like gene (*ENSSSCG00000014727*) was represented by four million reads (4% of the reads).

**Table 1:** Number of genes with sense and/or antisense transcription in the four tissues.

	JPP	IPP	MLN	PB
Expressed annotated genes	13,767	13,646	13,463	9,201
Number of genes that represented 50% of sense reads	648	619	719	2
Genes with antisense expression	3,155	3,138	3,616	1,345
Number of genes that represented 50% of antisense reads	38	34	43	1
Genes with both sense and antisense transcription	2,841 (21%)	2,836 (21%)	3,242 (24%)	1,059 (12%)
Genes harboring only antisense transcription	314	302	374	286

We quantified the transcription level of elements transcribed from the opposite strand of annotated genes in Ensembl (release 85) and found over 3,100 genes with measurable antisense transcription levels in PPs and MLNs, and less than half of that number in PB (Table 1). Globally, 4,138 loci annotated as genes on one strand in Ensembl were detected as transcribed on the other strand in at least one tissue.

As presented in Table 1, the GALT and PB showed an antisense transcription for over 21% and 12% of expressed genes, respectively. The five genes with the largest number of antisense overlapping reads were the same in all tissues: *GYS1* (*ENSSSCG00000003154*) with 48% of antisense reads in blood, *NDUFB10* (*ENSSSCG00000030328*), *RNF167* (*ENSSSCG00000017906*), *ND6* (*ENSSSCG00000018092*) and *FIB* (*ENSSSCG00000012969*). Of note, *NDUFB10* and *ND6* are both members of the *NADH*: ubiquinone oxidoreductase enzymatic complex. Besides, we detected an antisense transcription level for 598 non-expressed genes in our dataset (see full list in Supplementary Table S5). Interestingly, GALT shared 230 common annotated genes harboring only antisense transcription, and 101 antisense expressed events were shared by the four tissues.

We mapped the sense and antisense reads along the genome (see Supplementary Fig. S1 in Supplementary Information file). We observed that they are not homogeneously distributed, and that antisense transcription was preferentially localized on genomic regions showing sense transcription. Our results highlighted similar genomic regions across the four tissues that appear to be actively transcribed on both DNA strands.

We estimated correlations between sense and antisense gene transcription within and between tissues for the 14,596 genes with a CPM greater than 1 in at least one tissue and for the 4,138 genes with an antisense transcription level (CPM greater than 1 with at least 10 reads). The correlation of sense and antisense gene expression levels was found to be higher between IPPs and JPPs than between PPs and MLNs (Table 2). The correlation was lower between GALT and PB, and MLNs appeared to be more correlated with PB. No significant correlations were found between the antisense and sense read counts for a same gene, whatever the tissue.

**Table 2:** Spearman correlation coefficients of gene expression (below diagonal) and antisense transcription (above diagonal) between tissues; Spearman correlation coefficients between sense and antisense gene transription in each tissue (diagonal).

Antisense transcription	MLN	IPP	JPP	PB
Gene expression				
MLN	*−0*.*04*	0.72	0.72	0.71
IPP	0.83	*−0*.*06*	0.84	0.56
JPP	0.84	0.97	*−0*.*06*	0.58
PB	0.79	0.68	0.69	*−0*.*1*

### Differential gene expression analysis between GALT

**Figure 2:**
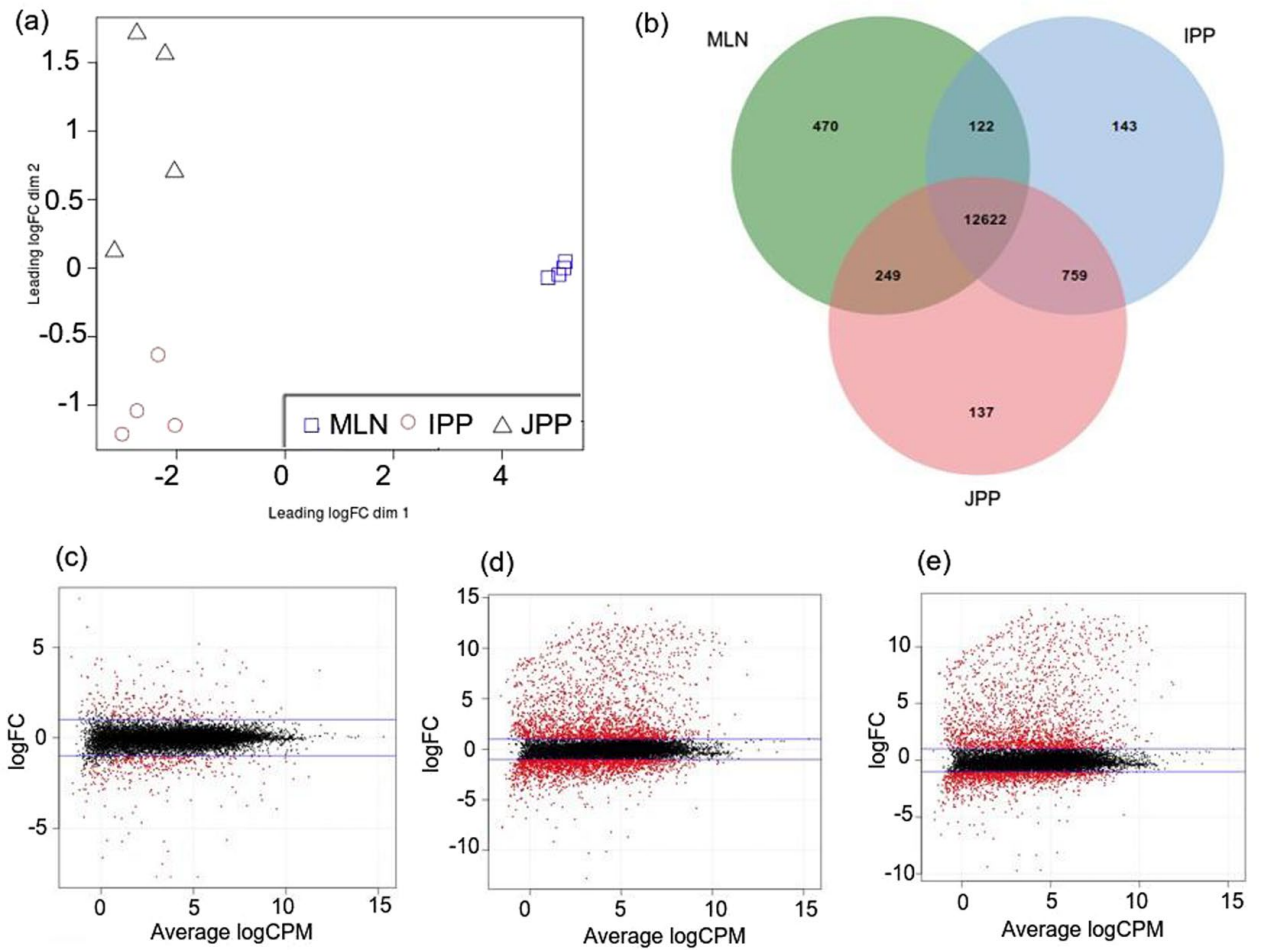
Summary plots of gene expression analyses within GALT. Graphs show an MDS plot for normalized gene expression levels for GALT (**a**), a Venn diagram for the comparison of expressed genes in GALT (**b**), and smear plots of differential gene expression analyses by comparing IPP and JPP (**c**), MLN and IPP (**d**) and JPP and MLN (**e**).

We performed a differential expression analysis within the GALT to explore differences in the transcriptome between PPs and MLNs as well as between IPPs and JPPs, based on the 14,502 genes found to be expressed (sense transcription with CPM > 1) in at least one of the three tissues (Fig. 2a). JPPs, IPPs and MLNs shared the same five most highly expressed genes: *cytochrome c oxidase subunit 1* (*COX-1*), *cytochrome c oxidase subunit 2* (*COX-2*), *cytochrome c oxidase subunit* 3 (*COX-3*), *ATP synthase F0 subunit 6* (*ATP6*) and *eukaryotic translation elongation factor 1 alpha 1 (EEF1A1)*. The *COX-1*, *COX-2* and *COX-3* mitochondrial genes, coding for cytochrome c oxidases, were represented by 8% and 5% of the sequencing reads in PPs and MLNs, respectively. *ATP6* is also a mitochondrial gene coding for an ATP synthase, and *EEF1A1* codes for a translation elongation factor.

In a first exploratory approach, an MDS plot for the GALT (Fig. 2b) showed that IPP and JPP samples were separated by intestinal segment, revealing differences in gene expression between IPPs and JPPs. We subsequently tested whether gene expression was significantly different between the GALT tissues using the edgeR package (Table 3). The complete lists of DE genes is available in Supplementary Tables S6 to S8. There were six times more differences between MLNs and PPs than between IPPs and JPPs. However, since fewer DE genes were detected, JPPs seemed to be more similar to MLNs than IPPs. Smear plots showed that the bulk of genes was centered around a log-fold change of zero indicating that any composition bias between libraries was successfully removed (Fig. 2c–e). We observed that the scale of fold changes between MLNs and PPs was larger than between IPPs and JPPs.

**Table 3:** Differential gene expression among the GALT samples.

	FDR < 0.05			FDR < 0.05 and |log_2_FC| > = 1		
	DE	Over-expressed	Under-expressed	DE	Over-expressed	Under-expressed
IPP VS MLN	6,852	3,405	3,447	3,922	2,143	1,779
JPP VS MLN	6,110	3,058	3,052	3,255	2,008	1,247
IPP VS JPP	1,103	444	659	549	217	332

**Figure 3:**
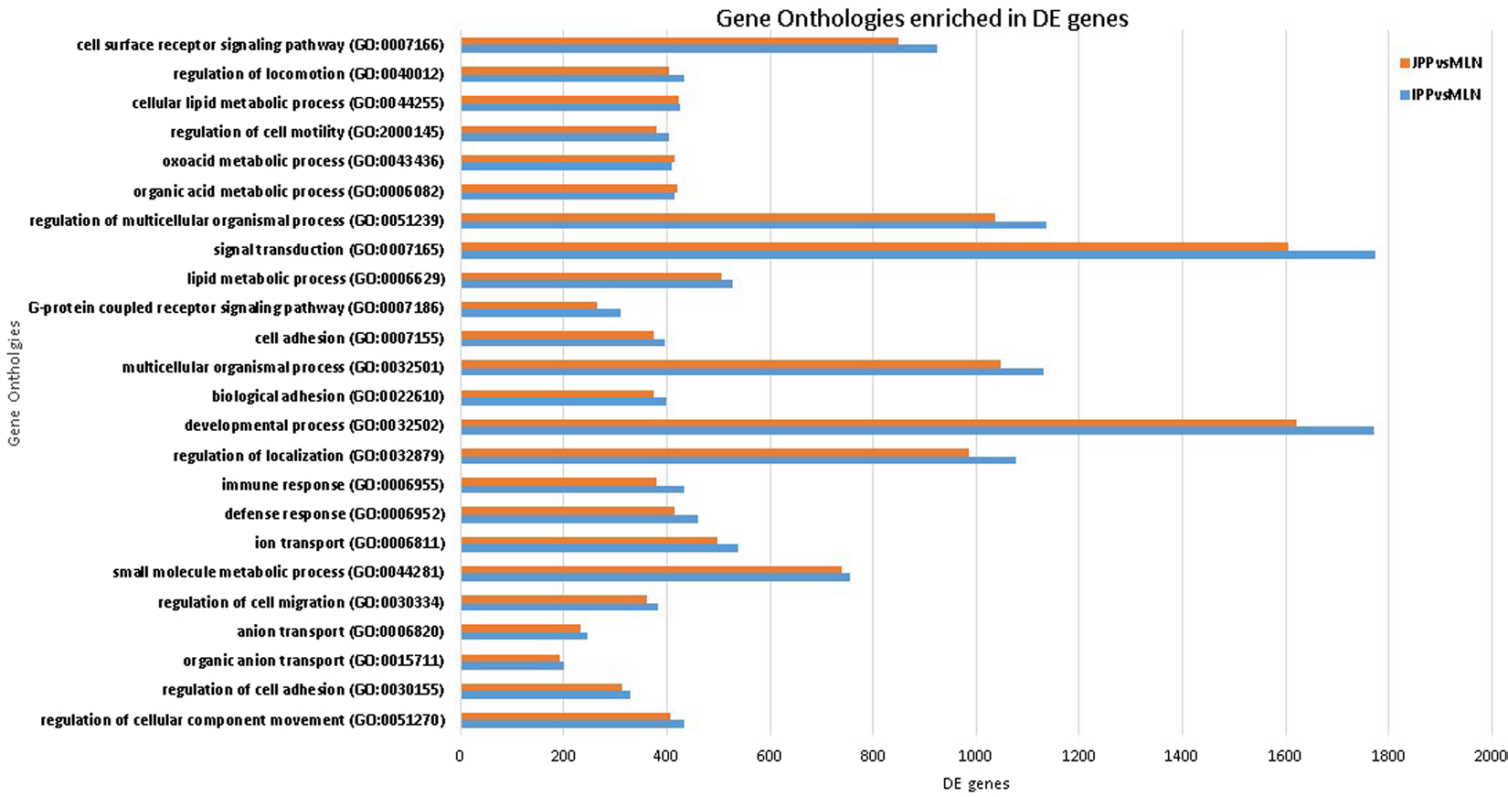
The most significantly enriched biological processes associated with differentially expressed genes between JPP and MLN (red) and IPP and MLN (in blue), performed with the GOrilla tool on human ortholog GeneNames. The bars represent the number of genes found DE involved in a given biological process, without taking into account whether the gene is under- or over-expressed in the comparison. The full lists of under- and over-expressed DE genes are available in Supplementary Tables S6–8.

**Figure 4:**
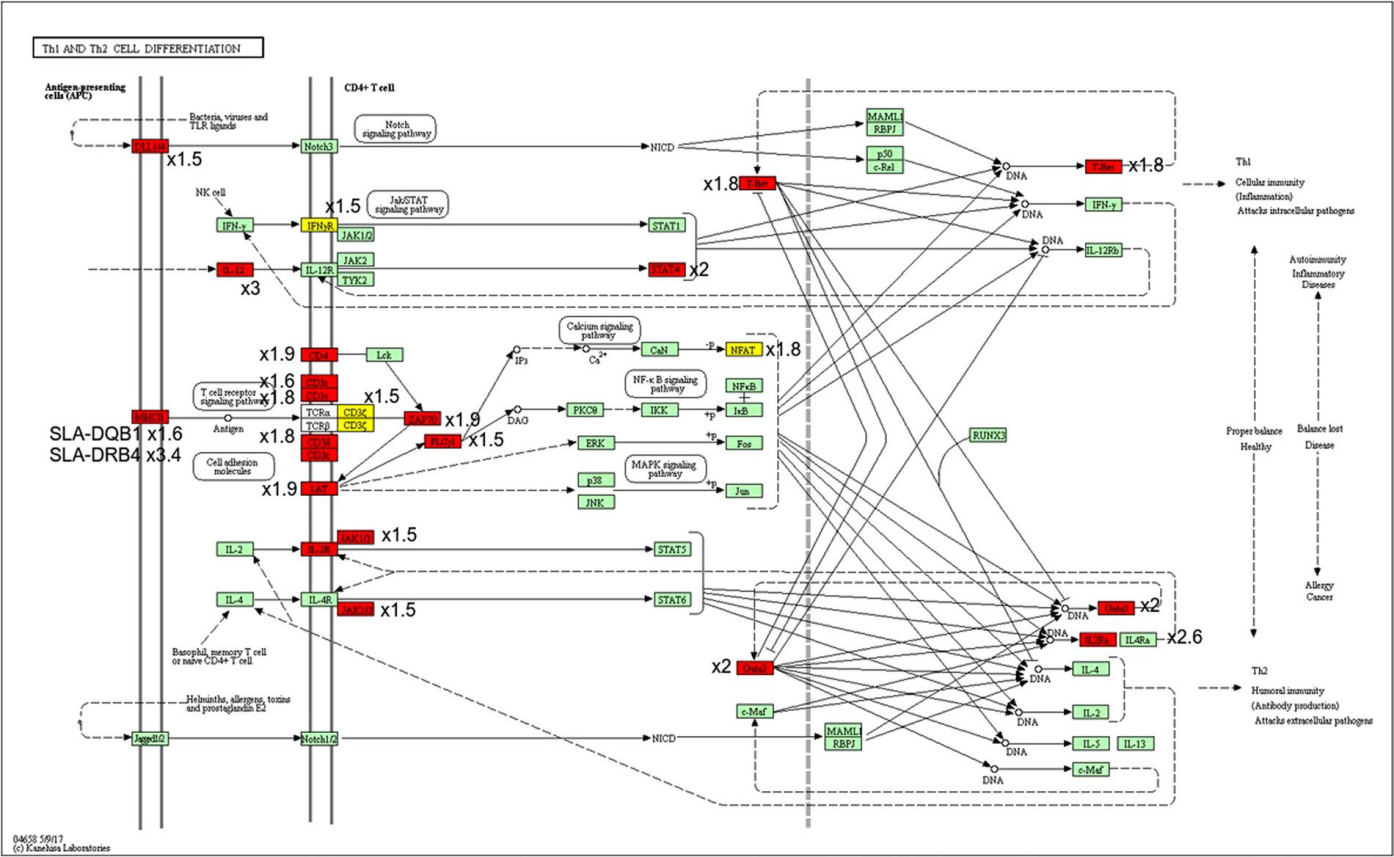
Th1 and Th2 cell differentiation pathways found enriched in DE genes between IPP and JPP by KEGG^25^. Genes over-expressed in IPP are shown in yellow and those over-expressed in JPP in red. The log-fold changes of each DE gene have been added to the figure.

DE genes between the PPs and MLNs were involved in cell surface receptor signaling pathway (GO:0007166) and signal transduction (GO:0007165). They were also involved in immune response (GO:0006955). They were implicated in cell adhesion (GO:0007155) and regulation of cell adhesion (GO:0030155), regulation of locomotion (GO:0040012) and regulation of cell motility (GO:2000145). They also affected metabolic processes such as lipid (GO:0006629) or organic metabolism processes (GO:0006082) (Fig. 3). The DE genes between IPPs and JPPs were found to be involved in 60 pathways referenced in the KEGG database^25^, where each of these pathways contained more than ten DE genes (Supplementary Table S9). The pathway that included the most DE genes (106) was “Metabolic pathway”, the second (44 DE genes) was “Pathways in cancer”, and the third (40 DE genes) was “Cytokine-cytokine receptor interaction”. The “Th1 and Th2 cell differentiation” pathway was also enriched and included 19 DE genes, of which 18 and one were under- and over-expressed in IPPs compared to JPPs, respectively (Fig. 4).

By comparing these results to our previous study that targeted transcriptome analysis along the small intestine (duodenum, jejunum, ileum)^12^, we found an overlap of 431 genes between the set of DE genes previously identified between jejunum and ileum and the 1,105 DE genes between IPPs and JPPs. The fold change ranges were quite similar in both studies (respectively 1.7 versus 1.9 log_2_FC), and 97% of the shared genes showed a similar trend of differential expression, with only 14 genes presenting contradictory differential expression patterns between ileum/jejunum and IPPs/JPPs. The 431 shared DE genes were involved in pathways referred to as “LPS/IL-1 Mediated Inhibition of RXR Function”, “FXR/RXR Activation”, and “LXR/RXR Activation”. The top ten DE genes in term of absolute log-fold change most DE genes were assembled by IPA in various networks related to lipid metabolism (Supplementary Table S10), as reported in Mach *et al*.^12^. In addition, over 60% of the DE genes (672 genes) between both PP types were not found DE in the intestinal segments in Mach *et al*.’s results^12^. In enrichment analyses, DE genes specific to lymphoid tissues were found to be involved in several immunity-related canonical pathways: “Th1 and Th2 Activation Pathway”, “Primary Immunodeficiency Signaling”, “Th1 Pathway”, “iCOS-iCOSL Signaling in T Helper Cells”, and “T Cell Receptor Signaling”. The most enriched functions were “Hematological System Development and Function”, “Tissue Morphology”, and “Lymphoid Tissue Structure and Development”. The top ten DE genes between IPPs and JPPs assembled in IPA networks were related to biological functions linked to lymphoid tissue structure, cellular development and cell-mediated immune response.

### Allelic specific expression analyses in porcine GALT and blood transcriptomes

Beyond the observed differential expression patterns between the GALT, we further explored the *cis*-genetic control of gene expression in each tissue using an ASE analysis. Overall, 432,198 SNPs were called across the 16 samples (MLN, IPP, JPP and PB from four individuals), after filtering. Each animal was genotyped according to the RNA-Seq data for the four tissues with the QuASAR R package^22^, and 157,178 SNPs were found to be heterozygous in at least one animal. While 46% of the SNPs were shared by at least two pigs and 5,600 SNPs were heterozygous for the four pigs, each animal still harbored a large number of specific heterozygous SNPs (17,224 to 25,160).

For each tissue, we tested SNPs heterozygous in at least one animal (Table 4) for possible ASE. In total, 157,178 heterozygous SNPs overlapping 12,755 genes were tested for ASE in at least one tissue (Supplementary Table S11). Results showed 16,797 significant ASE SNPs, 52% of which were found significant for every heterozygous animal (Table 4). Overall, 37% of the 12,755 genes tested had at least one SNP with ASE in at least one tissue (Table 4), and we found 2,509 genes affected by more than one heterozygous SNP ASE.

**Table 4:** Summary of ASE results obtained from RNA-Seq datasets for the four tissues analyzed in this study (MLN, IPP, JPP and PB).

TISSUE	HETEROZYGOUS SNPS	GENES^1^	ASE SNPs (% tested)	Consistent ASE SNPs^2^ (% ASE SNPs)	ASE Genes (% tested)	ASE Genes > 1 ASE SNP (% ASE GENES)
MLN	129,122	11,667	8,295 (6.4%)	4,046 (49%)	2,945 (25%)	1,543 (52%)
IPP	111,378	11,576	8,774 (7.9%)	4,265 (49%)	2,956 (26%)	1,524 (52%)
JPP	113,475	11,570	8,583 (7.5%)	4,161 (48%)	2,877 (25%)	1,420 (49%)
PB	40,584	7,410	3,339 (8.2%)	1,787 (54%)	1,254 (17%)	614 (49%)
Total	157,178	12,755	16,787 (11%)	8,746 (52%)	4,769 (37%)	2,509 (53%)

^1^Genes with a heterozygous SNP (<5 kb distance from gene according to Ensembl’s Variant Effect Predictor).

^2^For each animal heterozygote at this position, the SNP was detected with a significant ASE.

The proportion of genes showing significant ASE in at least one individual was close to 25% for GALT and 17% for blood. Of the genes showing significant ASE, 16% displayed ASE in the four tissues and were found to be involved in immune related functions such as antigen processing and presentation, T cell receptor signaling pathway of type I interferon signaling pathway by enrichment analyses. In PB, 141 genes were specifically under a *cis*-genetic control while they were also expressed in other tissues. In MLNs, 669 genes specifically displayed an ASE. By comparing MLNs and PPs, 113 genes expressed in GALT displayed specific ASE in PPs. 500 genes expressed in JPPs and 572 genes expressed in IPPs had ASE while expressed in the other tissues with no allelic imbalance (Supplementary Table S12). Nevertheless, the ASE genes specific to each of these tissues were not enriched in any functional category.

### Validation of differentially expressed genes and ASE signals

**Figure 5:**
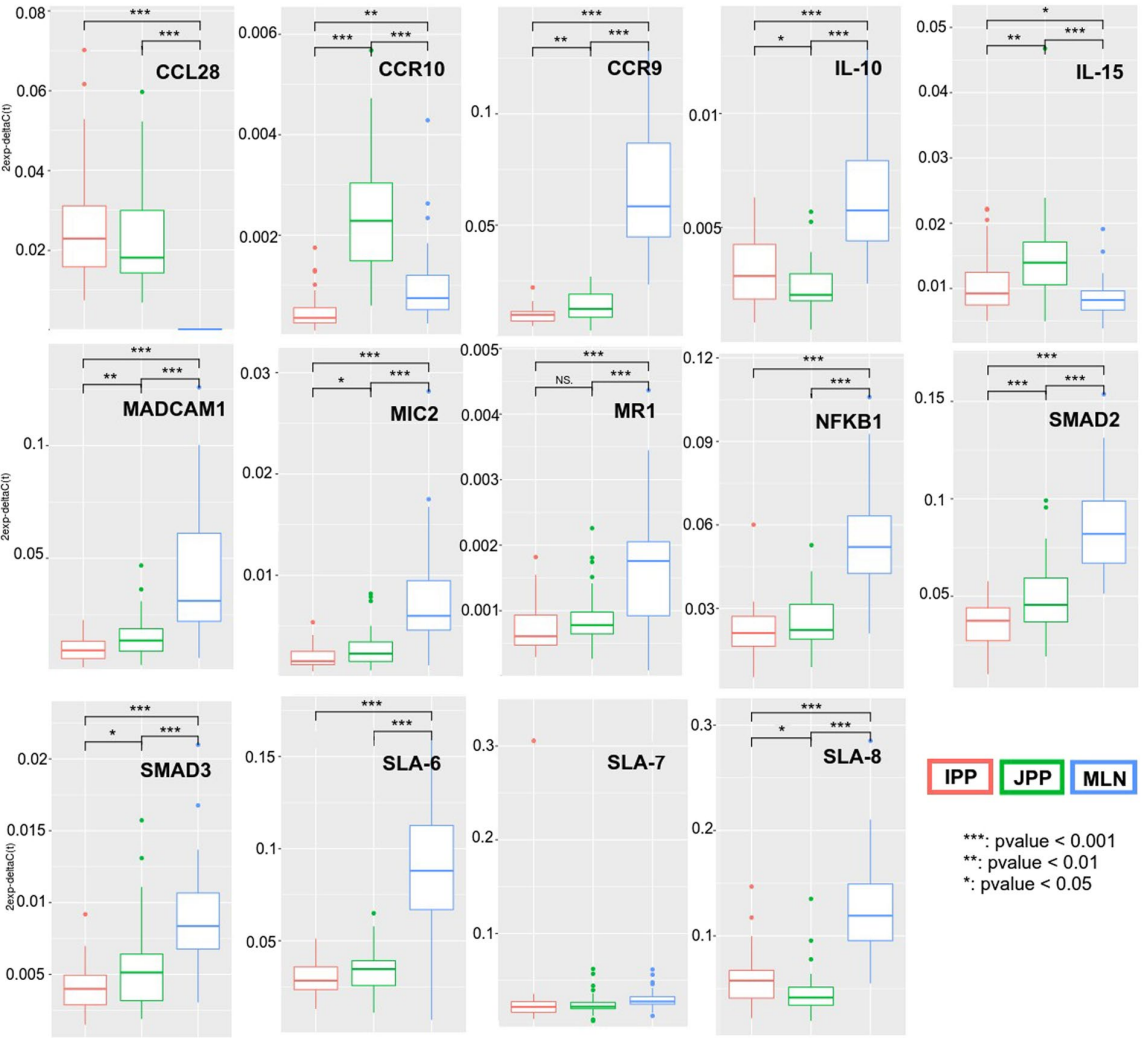
Validation of the differential expression of a subset of genes between IPP, JPP and MLN by qRT-PCR.

qRT-PCRs were carried out for 24 genes (Supplementary Table S3) using tissues collected from the group of 32 pigs of 70 days of age belonging to the same cohort as the four animals included in the transcriptome analysis. The results confirmed that the *CCR10* and *CCR9* genes were under-expressed in IPPs versus JPPs (Fig. 5 and Supplementary Table S13). Furthermore, we showed that *CCR10* was less expressed in IPPs compared to MLNs, but more highly expressed in JPPs compared to MLNs and IPPs. We also validated that *SLA-7* was not differentially expressed between GALT samples. qRT-PCRs confirmed that *CCL28* and *IL-15* were more expressed in PPs than in MLNs, and that *SLA-6*, *SLA-8*, *MIC-2*, *MR1*, *IL-10*, *CCR9*, *MADCAM1*, *NFKB1*, *SMAD2* and *SMAD3* were down-regulated in PPs in comparison to MLNs.

As a first validation step of the ASE results, we compared the current ASE analysis on blood with a previous study that we conducted on 38 60-day-old Large-White pigs on peripheral blood^13^. Here, we revealed 1,005 ASE-SNPs (FDR < 0.05, at least 10 reads) found in at least two heterozygous pigs, of which 39% of these SNPs (395) were already described as ASE-SNPs in our precedent study. Among them, 332 SNPs are described in dbSNP and include 55% missense variants, 43% synonymous variants and 1% stop gained variants. These 332 SNPs affected 93 genes.

In a second step, we used the pyrosequencing allele quantification approach to evaluate the consistency of the ASE effects detected in six genes and representing a range of allele frequencies and tissue specificities: *lysozyme* (*LYZ*), *immunoglobulin lambda variable* 10 (*IGVL10*), *complement factor D* (*CFD*), *C-X-C motif chemokine ligand 13* (*CXCL13*), *2*,*−5*,*-oligoadenylate synthetase 2* (*OAS2*) and *germinal center-associated*, *signaling and motility* (*GCSAM*). Out of these six genes, we validated the RNA-Seq results in three of the cases representing a tissue-specific ASE (*LYZ*, with ASE confirmed only in MLNs and PB) and two strong ASE effects (*IGLV10* and *OAS2*). However, ASE effects of smaller magnitude could not be confirmed for *CFD*, *CXCL13* and *GCSAM* (Supplementary Table S14).

## Discussion

Improving individual health in pig production systems requires a good understanding of immune and intestine pig functions to foster new disease preventive approaches. The pig species is also considered to be an excellent model for biomedical research, particularly for intestinal diseases due to anatomical and physiological similarities with the human digestive system^31^. However, swine PPs are an exception to this rule because, as in ruminants and rabbits, they have the particularity of forming a continuous structure in the ileum, while in humans they constitute independent follicles in all intestinal segments as in the pig jejunum^31,30,32^.

We report here a comparative analysis of the transcriptomic profiles of GALT in pigs including IPPs, JPPs and MLNs, and PB as non-GALT reference immune tissue. The results showed extensive expression differences across GALT that were likely to reflect tissue specificities. The mapping of the *cis-*genetic control of gene expression with ASE analyses uncovered both shared and specific ASE signals in each tissue. Overall, our results propose a global picture of the transcriptomic control and profiles of GALT tissues in 70-day-old pigs in good health as a reference study to pave the way for a better understanding of the molecular mechanisms of PP functions in pigs.

The porcine IPPs and JPPs have transcriptomic specificities linked with immunity. The anatomical differences between IPPs and JPPs in pigs have been previously described^4,33^, and several studies have reported the transcriptome profiles of JPPs^34,35^. However, to our knowledge, comparative analyses of gene expression between JPPs and IPPs in pigs have only been investigated in limited sets of target immune response-related-genes^10,11^. Thus, our study is the first whole-transcriptome approach to this question, revealing 1,103 DE genes between IPPs and JPPs in pigs. Since, to the best of our knowledge, there are not equivalent reports on ruminants like sheep and cattle, our results could also be relevant for these species. The differential gene expression profiles between the two PP types could be partly related to their anatomical position along the small intestine. We thus compared the IPP and JPP transcriptomes to those of the ileum and jejunum sections previously reported^12^. We confirmed that genes found DE between IPPs and JPPs represented specific differences between the two PP types and were not simply reflecting the gene expression profiles of their corresponding intestinal segment.

We analyzed the gene sets found DE between IPPs and JPPs by performing IPA-based enrichment studies in order to explore which pathways were affected. We observed that canonical pathways related to T cell activity were down-regulated in IPPs while the B cell receptor-signaling pathway was up-regulated in IPPs compared to JPPs. These differences were consistent with the variations in lymphocyte composition described in pigs, since IPPs were reported to contain more B cells than JPPs (90% and 45% of B lymphocytes, respectively), and fewer T cells^7^. Several studies have described that the relative number of cells entering the IPPs was lower than that entering the JPPs in pigs^36,37^. Accordingly, we found that the receptor *CCR7* and its ligand *CCL21* that mediate the organization of the T zone^38^ were less expressed in IPPs. The *CCR10* gene required for optimal T cell dependent IgA-antibody secreting cells accumulation in mice^39^ was also found to be less expressed in the IPPs (logFC = −1.3). Furthermore, the receptor *CTLA4*, involved in the regulation of activated T-lymphocytes^40^, was found to be less expressed in the IPPs, and *CCR9*, which promotes the homing of IgA-antibody secretion T cells towards the intestine^41^, was found more expressed in JPPs (logFC = 0.8) than in IPPs as described previously^10^. Thus, all together our results are consistent with a higher proportion of T cells in the JPPs compared to the IPPs.

Our results highlighted gene expression differences between IPPs and JPPs related to the differentiation and fate of T cells (see Supplementary Table S9). The master regulators of CD4+ T cell commitment and plasticity have been extensively studied^42^. A slight but significant decrease in the master regulators known to be involved in naïve CD4+ T cell differentiation was observed in IPPs compared to JPPs (FC between −1.8 and −2.6 and logFC between −0.87 and −1.42 see Fig. 4). The transcription factors found less expressed in IPPs were *FOXP3* (differentiation of induced T regulator cells or iTreg), *GATA3* (Th2 differentiation), *STAT4* and *TBX21* that encodes T-bet (Th1 differentiation), as well as *RORC* that encodes Rorγt (Th17 differentiation). Conversely, the transcription factor *BCL6* was found over-expressed in IPPs compared to JPPs (FC = 1.8 and logFC = 1.83). *BCL6* is involved in the differentiation of the follicular helper T (Tfh) cells that have the important function of providing B-cell help for the induction of antigen-specific antibody production^43^. The Tfh cells contribute to the formation of germinal centers, which are dynamic microenvironments that provide a unique niche for B-cell affinity maturation to occur (review in Ramiscal and Vinuesa)^44^. *ICOS*, known to be expressed on surface of Tfh cells, was however found less expressed in IPPs (FC = −2.6 and logFC = −1.4) than in JPPs as well as *IL-21* (FC = −2.8 and logFC = −1.5), known to be produced by Tfh cells^43^. It has been reported in mice that a fraction of Foxp3+ T cells can become Tfh cells in the PPs after down-regulating *FOXP3* expression^45^. This finding is in agreement with our results that revealed opposite differential expression between *FOXP3* and *BCL6* in IPPs and JPPs. Both JPPs and IPPs are main sites for priming B cells and educating the adaptive immune system while facing the gut microbiota. Tfh cells seem to play a crucial role in sensing microbiota-derived extracellular ATP and shaping commensal microbiota composition^46^. Our results suggest that IPPs are more abundant in Tfh and B cells than JPPs, and this could be linked to the densification of the microbiota along the digestive tract. Even if we cannot exclude that the differential expression found between IPPs and JPPs could be solely due to distinct proportions of B and T cells, our results also suggested that T cells could have different fates in IPPs and JPPs.

Regarding the coherence of our results with previous reports, we observed that *CCR7-CCL21*, which mediate the organization of PPs into follicles, were down-regulated in the IPPs. Moreover, we found that the *TNFSF11* gene involved in the development of GALT was less expressed in IPPs in comparison with JPPs (logFC < −1). These results are consistent with differences in the development kinetics between JPPs and IPPs as reported in two month old piglets, with a slower development of IPPs compared to JPPs^47^. In addition, Gourbeyre *et al*.^11^ highlighted differential expression in JPPs and IPPs for *TLR2*, *TLR6*, *TLR7*, *TLR9*, *TLR10*, and *NLRP3*. We also observed a differential expression of *TLR7* and *TLR8* (IPPs < JPPs) and *TLR9* (IPPs > JPPs).

MLNs and PPs participate in the activation of lymphocytes with the presentation of antigens passing through the intestine^48^. Interestingly, *TNFSF11*, a gene involved in the development of lymph nodes and not of early steps in the PP development, was found under-expressed in PPs in comparison with MLNs. Boeker *et al*.^33^ explored the distribution of the major lymphocyte populations in the peripheral blood and lymphoid organs in six female Göttingen minipigs. Our results were consistent with their immune-histological data. Indeed, we observed an under-expression of the chemokines *CXCL12*, *CXCL13*, *CXCR5*, and *CX3CR1* that mediate the attachment of cells to the lymphoid organs in PPs by comparison with MLNs.

Beyond describing the differences in gene expression within GALT, we studied the regulation of gene expression by performing antisense transcript and ASE analyses in MLNs, JPPs, IPPs and PB. We did not intend here to predict new antisense transcripts in intergenic regions, but only to quantify antisense transcription in annotated genes. Similarly to the work of He *et al*.^49^ on five different cell types, we observed that antisense transcription was not homogenous along the genome. Antisense transcription also seemed to be more frequent in regions with sense transcription. This might be due to a density reduction of the nucleosome structure where genes are transcribed, which may facilitate genome accessibility for antisense transcription. Chen *et al*.^50^ studied antisense transcription in liver and muscle of a pig F2 generation. They observed that 26% to 32% of liver transcripts and 46% to 54% of muscle transcripts had an antisense transcription, respectively. In our study, we found 24%, 22% and 14% genes with antisense transcription in MLNs, PPs and PB, respectively. The absence of significant correlations between sense and antisense transcription levels suggest that sense and antisense transcriptions are not co-regulated and are subjected to distinct regulation mechanisms.

Regarding ASE, we found several effects across the four tissues (Table 4) and the enrichment analyses showed an overabundance of immune-related functions associated with the genes shared between the four tissues. Overall, 37% of the tested genes displayed an ASE effect in at least one tissue, indicating the extensive *cis*-regulation present in the immunome. A recent study conducted by Chamberlain *et al*.^51^ in cattle showed even more *cis*-regulation, with 89% of genes harboring heterozygous SNPs having at least one SNP with significant ASE. Similarly, Crowley *et al*.^52^ reported that 89% of all genes tested in mouse brain show ASE.

We observed an important specificity in the *cis*-genetic control of gene expression between MLNs and PPs, although intriguingly there were not specific enriched functions associated with these genes. For individual tissues, the proportion of genes showing significant ASE is close to 25% for GALT and 17% for blood. These proportions are in agreement with Chamberlain *et al*.^51^ who previously reported percentages of genes showing significant ASE for white blood cells (33%), spleen (12%) and intestinal lymph node (31%) in cattle. The GTEx consortium^53–55^ found that between 1.5 and 3.7% of 6,385 tested SNPs showed ASE; here we found globally 11% of tested SNPs showed an ASE, confirming a likely significant proportion of regulatory SNPs in the pig genome^13^.

Since the blood transcriptome is widely used as a source of biomarkers, we have performed global correlation analyses to evaluate whether it recapitulates at least in part the transcriptomes of GALT. The PPs, MLNs and PB are complex multicellular tissues, and their gene expression profiles reflect the transcription levels of multiple cell types with an expected over-representation of genes expressed in the most abundant cell subset. As expected, transcripts from PB were dominated by the major blood proteins (hemoglobin and ferritin) and reduced the number of low expression genes that could be detected. Nevertheless, we observed strong positive correlations between the transcription levels of genes expressed in blood and GALT for both sense and antisense transcripts (Table 2), despite the different time of sampling between blood and GALT. These data support the use of blood in pigs as a surrogate tissue and a potent source of biomarkers for functions taking place in other tissues. In addition, we found considerable overlap of ASE effects between GALT and blood, highlighting a shared *cis*-genetic control for the expression of immunity-related genes.

## Electronic supplementary material

Supplementary Information

Supplementary Table S1

Supplementary Table S2

Supplementary Table S3

Supplementary Table S4

Supplementary Table S5

Supplementary Table S6

Supplementary Table S7

Supplementary Table S8

Supplementary Table S9

Supplementary Table S10

Supplementary Table S11

Supplementary Table S12

Supplementary Table S13

Supplementary Table S14
