# Supplementary material for: Immunome differences between porcine ileal and jejunal Peyer’s patches revealed by global transcriptome sequencing of gut-associated lymphoid tissues
Source: Sci Rep. 2018 Jun 13;8:9077. doi: 10.1038/s41598-018-27019-7 (PMC5998120; doi:10.1038/s41598-018-27019-7)
Supplement: Supplementary file 1 — Supplementary Information [file 41598_2018_27019_MOESM1_ESM.pdf]

Supplementary Information

Immunome differences between porcine ileal and jejunal Peyer's patches revealed by global transcriptome sequencing of gut-associated lymphoid tissues

T. Maroilley[1], M. Berri[2], G. Lemonnier[1], D. Esquerré[3], C. Chevaleyre[2], S. Mélo[2], F. Meurens[2,#], J.L. Coville[1], J.J. Leplat[1,4], A. Rau[1], B. Bed'hom[1], S. Vincent-Naulleau[4], M.J. Mercat[5], Y. Billon[6], P. Lepage[7], C. Rogel-Gaillard[1,*] and J. Estellé[1]

[1] GABI, INRA, AgroParisTech, Université Paris-Saclay, 78350 Jouy-en-Josas, France

[2] ISP, INRA, Université Tours, 37380, Nouzilly, France

[3] GenPhySE, INRA, INPT, ENVT, Université de Toulouse, 31326 Castenet-Tolosan, France

[4] LREG, IRCM, DRF, CEA, Université Paris Saclay,78350 Jouy-en-Josas, France

[5] BIOPORC and IFIP-Institut du porc, La Motte au Vicomte, BP 35104, 35651 Le Rheu, France

[6] GENESI, INRA, 17700 Surgères, France

[7] MICALIS Institute, INRA, AgroParisTech, Université Paris-Saclay, 78350 Jouy-en-Josas, France

*Corresponding author

[#] Current address: BIOEPAR, INRA, Oniris, La Chantrerie, 44307, Nantes, France

Supplementary Figure S1: Density of sense and antisense reads aligned along the reference genome

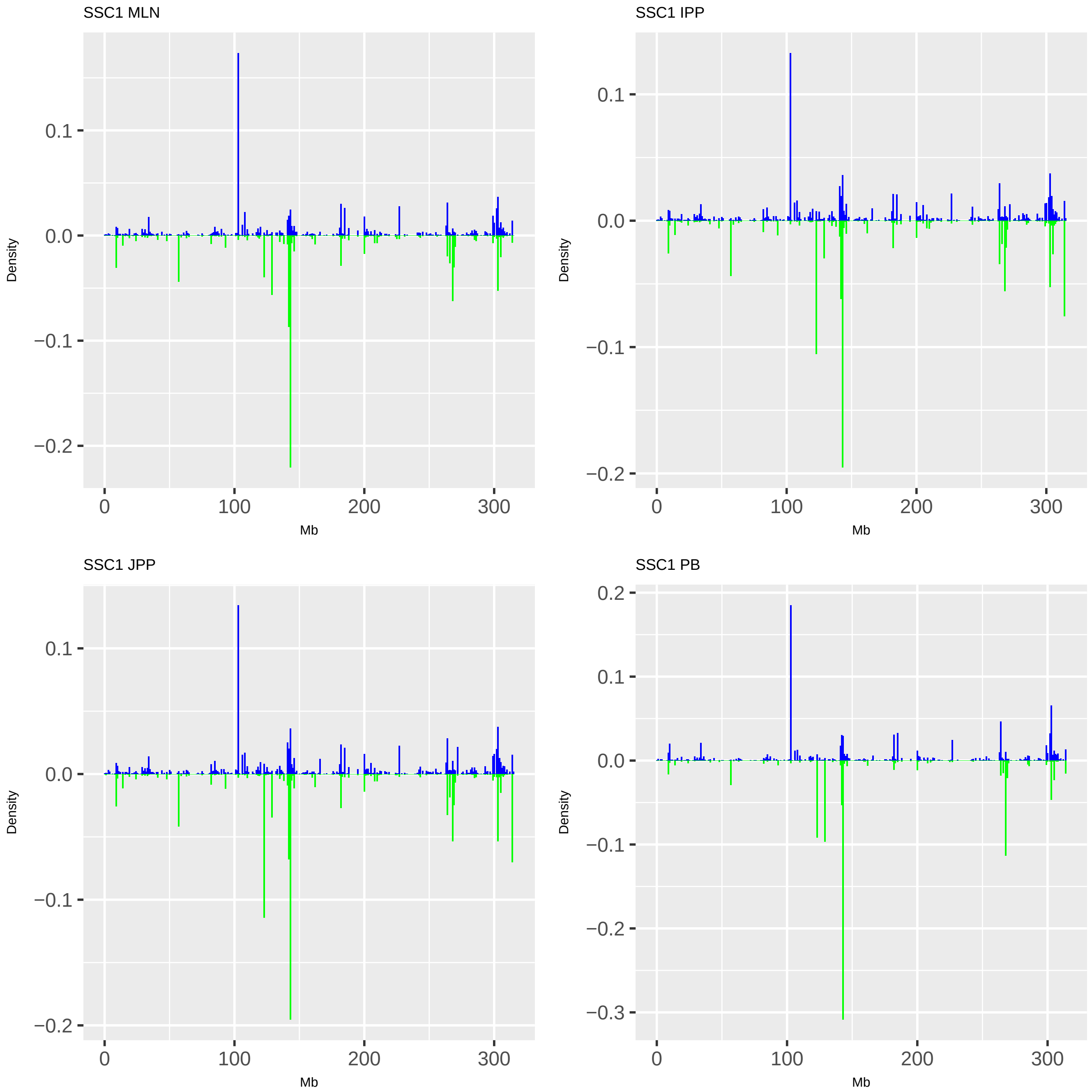
SSC1 MLN
SSC1 IPP
SSC1 JPP
SSC1 PB
Density
Mb
0.1
0.0
−0.1
−0.2
0.2
−0.3
0
100
200
300

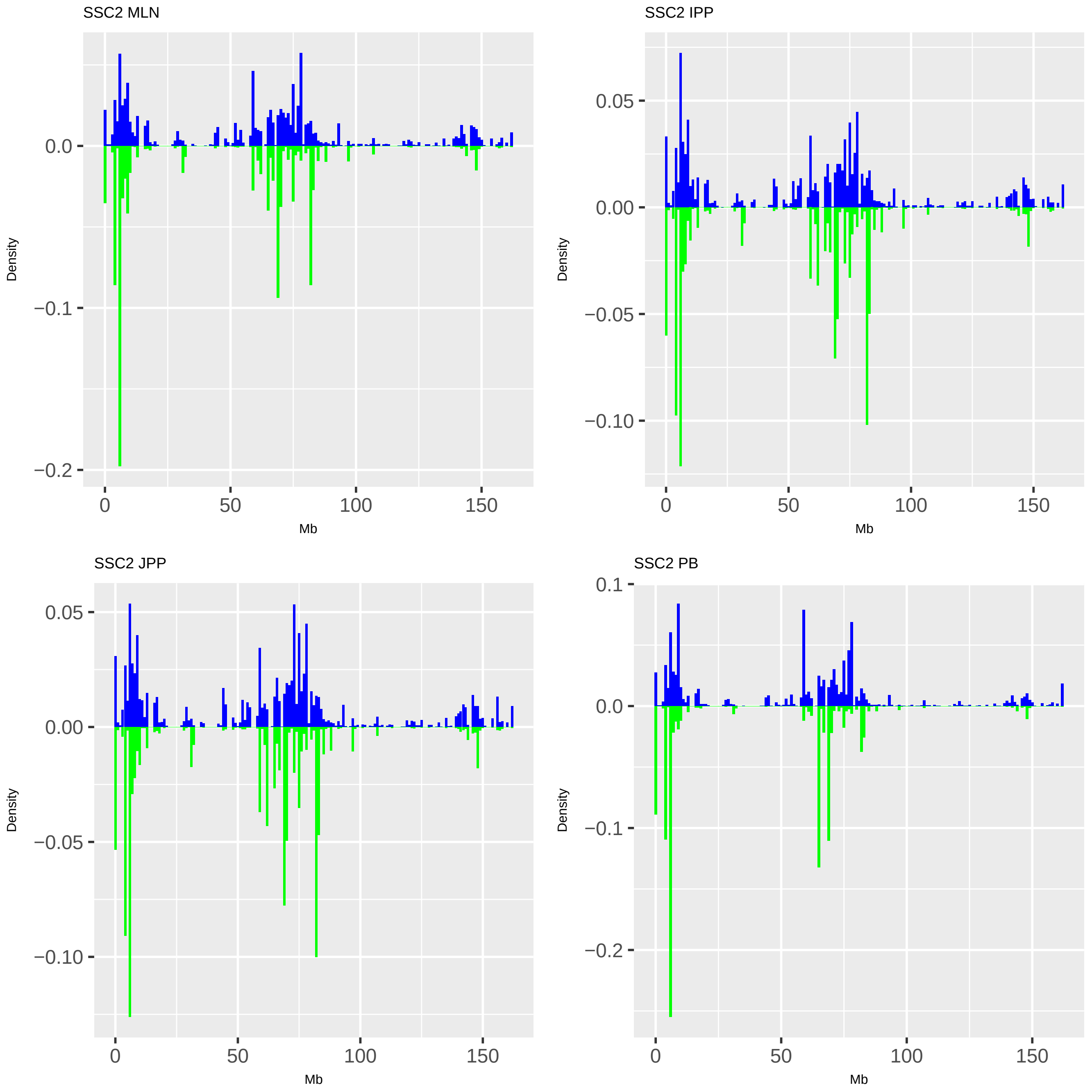
SSC2 MLN
SSC2 IPP
SSC2 JPP
SSC2 PB
Density
Mb

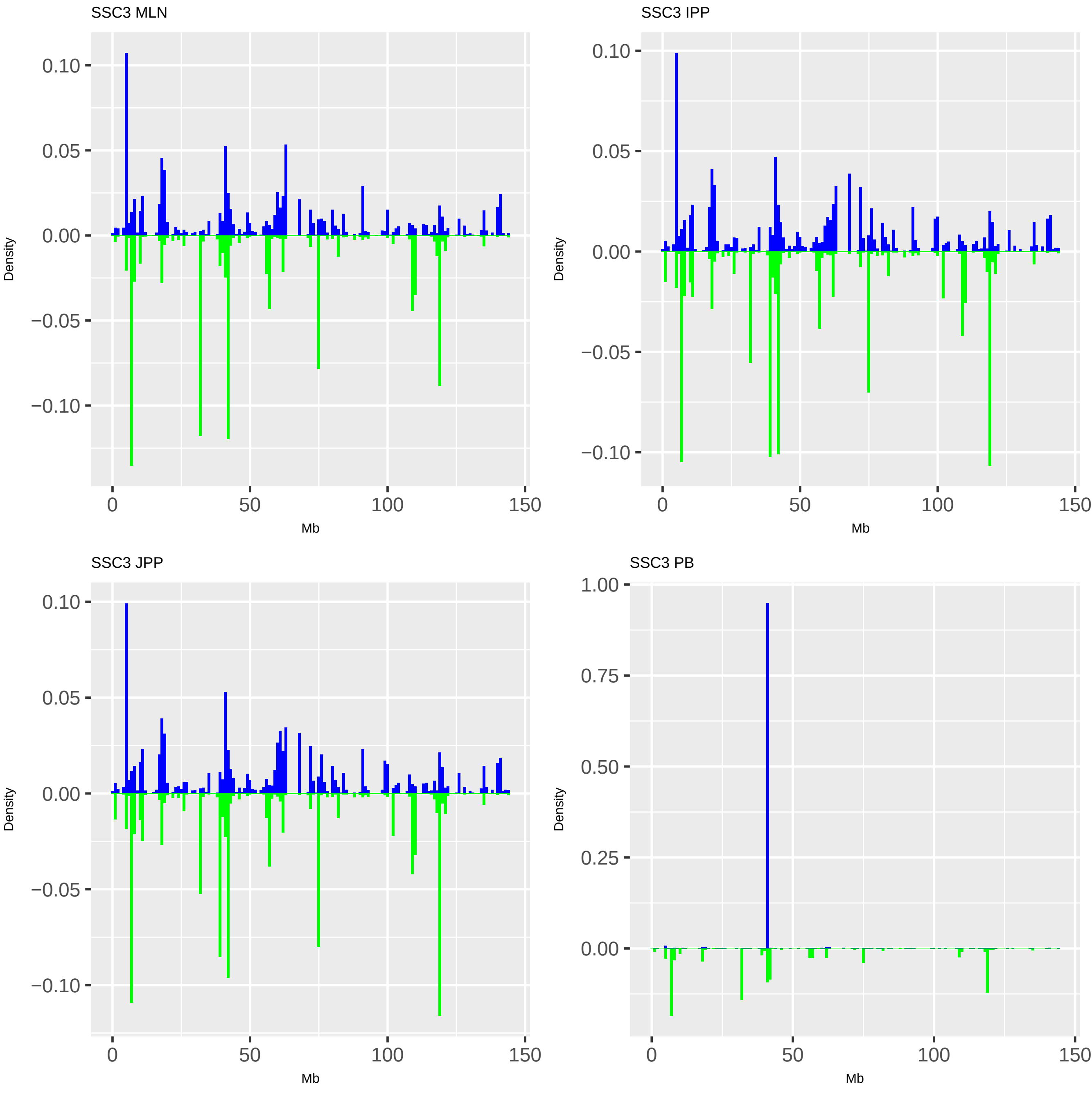
SSC3 MLN
SSC3 IPP
SSC3 JPP
SSC3 PB
Density
Mb
0.10
0.05
0.00
−0.05
−0.10
1.00
0.75
0.50
0.25
0
50
100
150

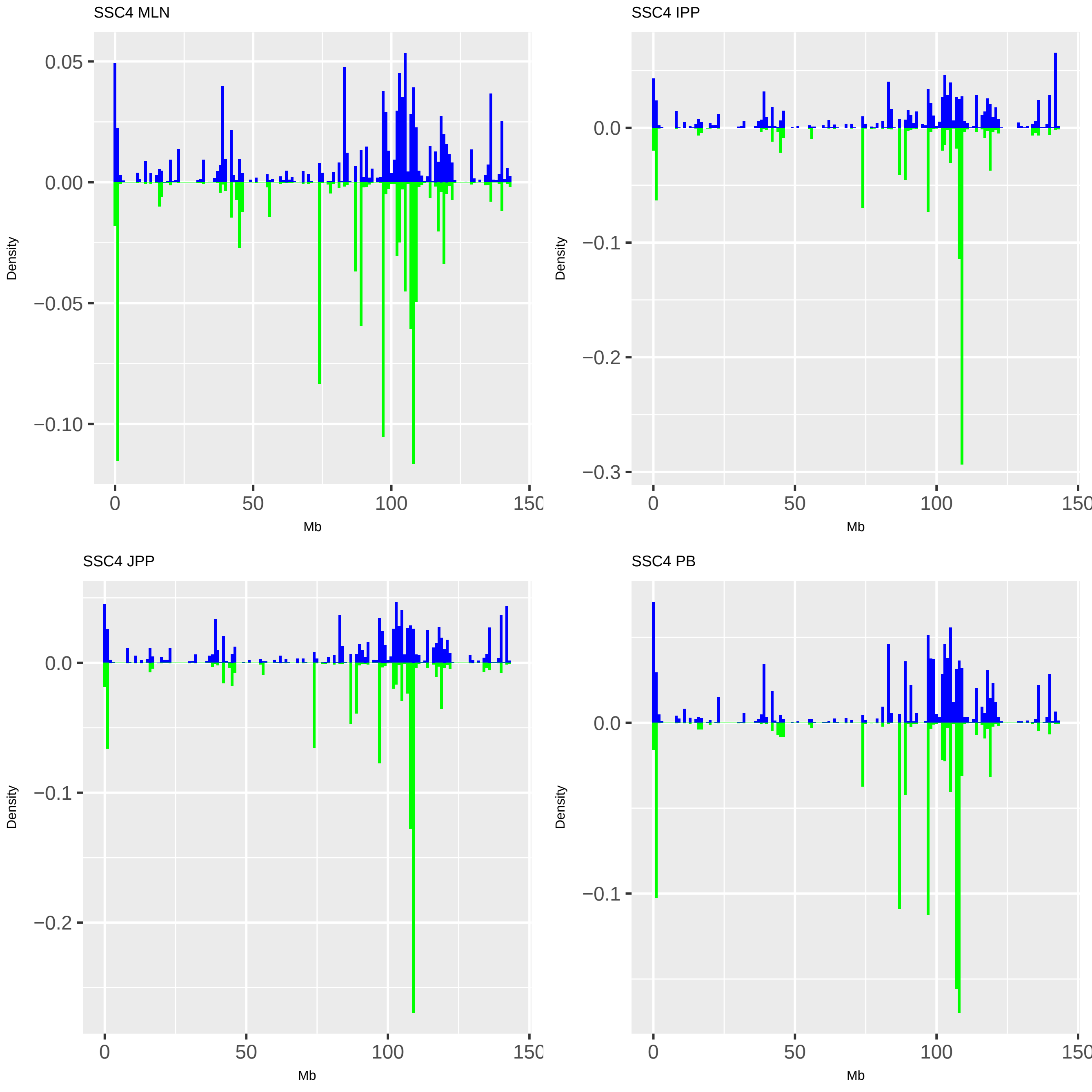

SSC4 MLN
SSC4 IPP
SSC4 JPP
SSC4 PB
Density
Mb
0.05
0.00
−0.05
−0.10
0.0
−0.1
−0.2
−0.3
0
50
100
150

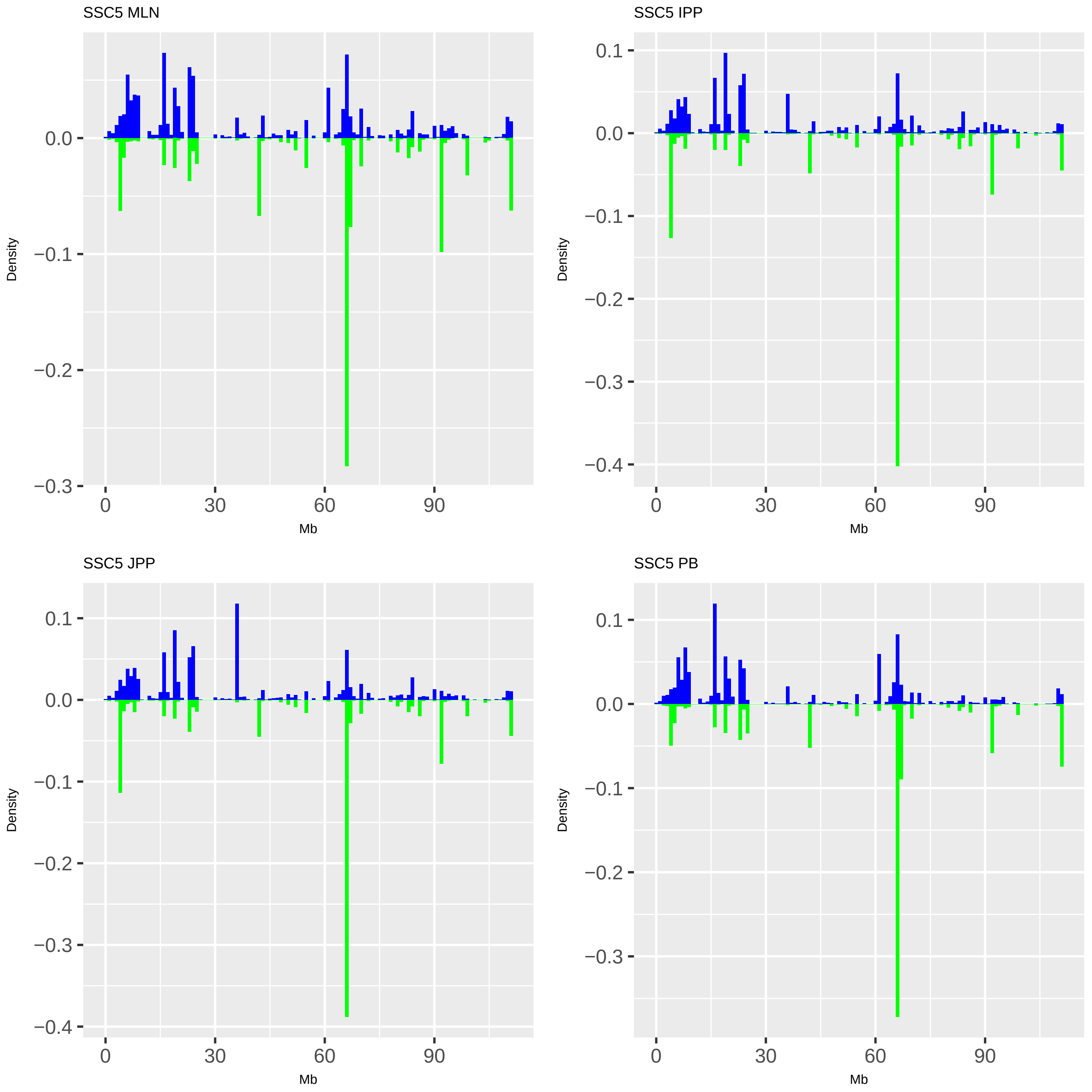
SSC5 MLN
SSC5 IPP
SSC5 JPP
SSC5 PB
Density
Mb
0.1
0.0
−0.1
−0.2
−0.3
−0.4
0
30
60
90

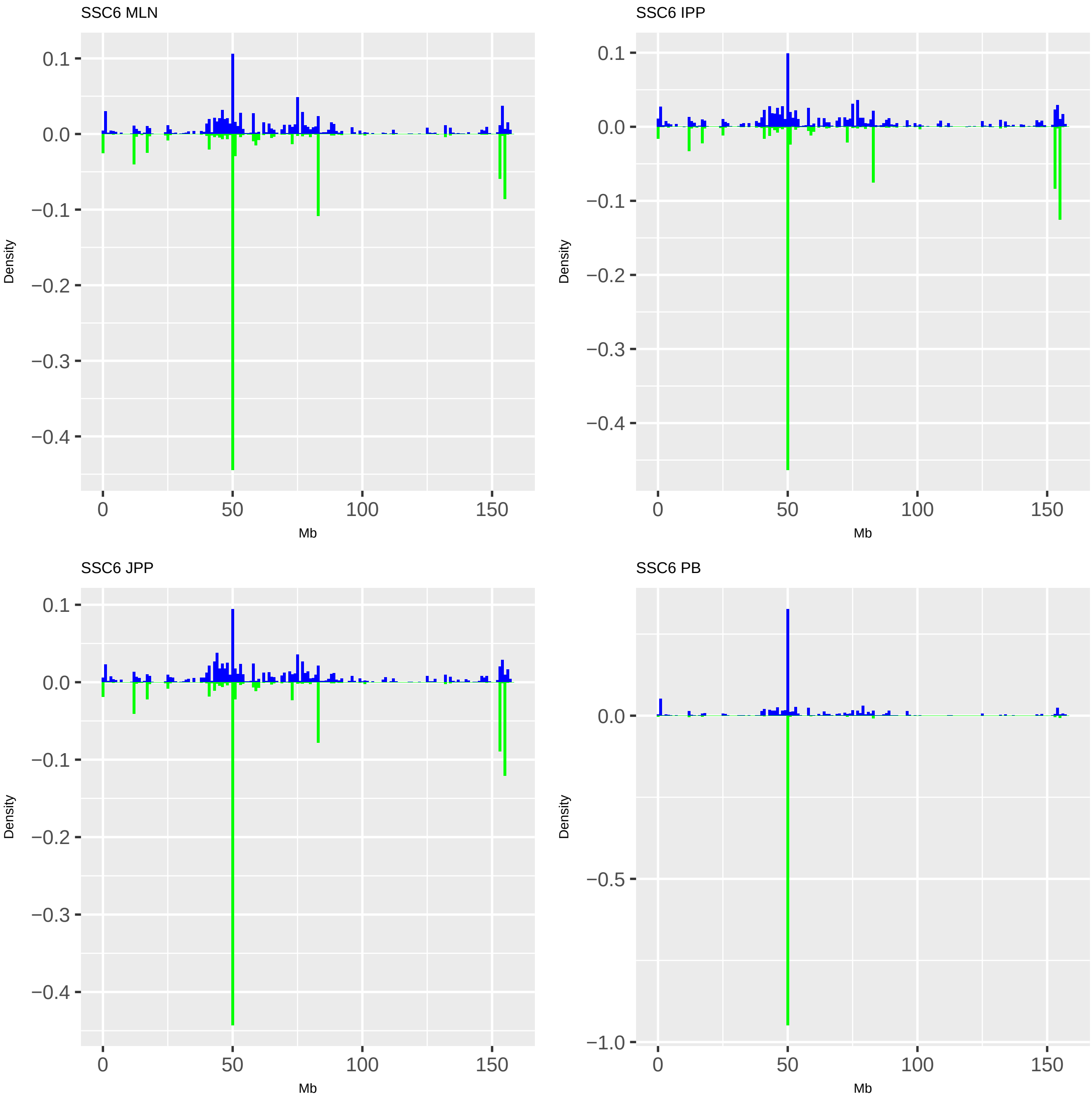
SSC6 MLN
SSC6 IPP
SSC6 JPP
SSC6 PB
Density
Mb
0.1
0.0
−0.1
−0.2
−0.3
−0.4
−0.5
−1.0
0
50
100
150

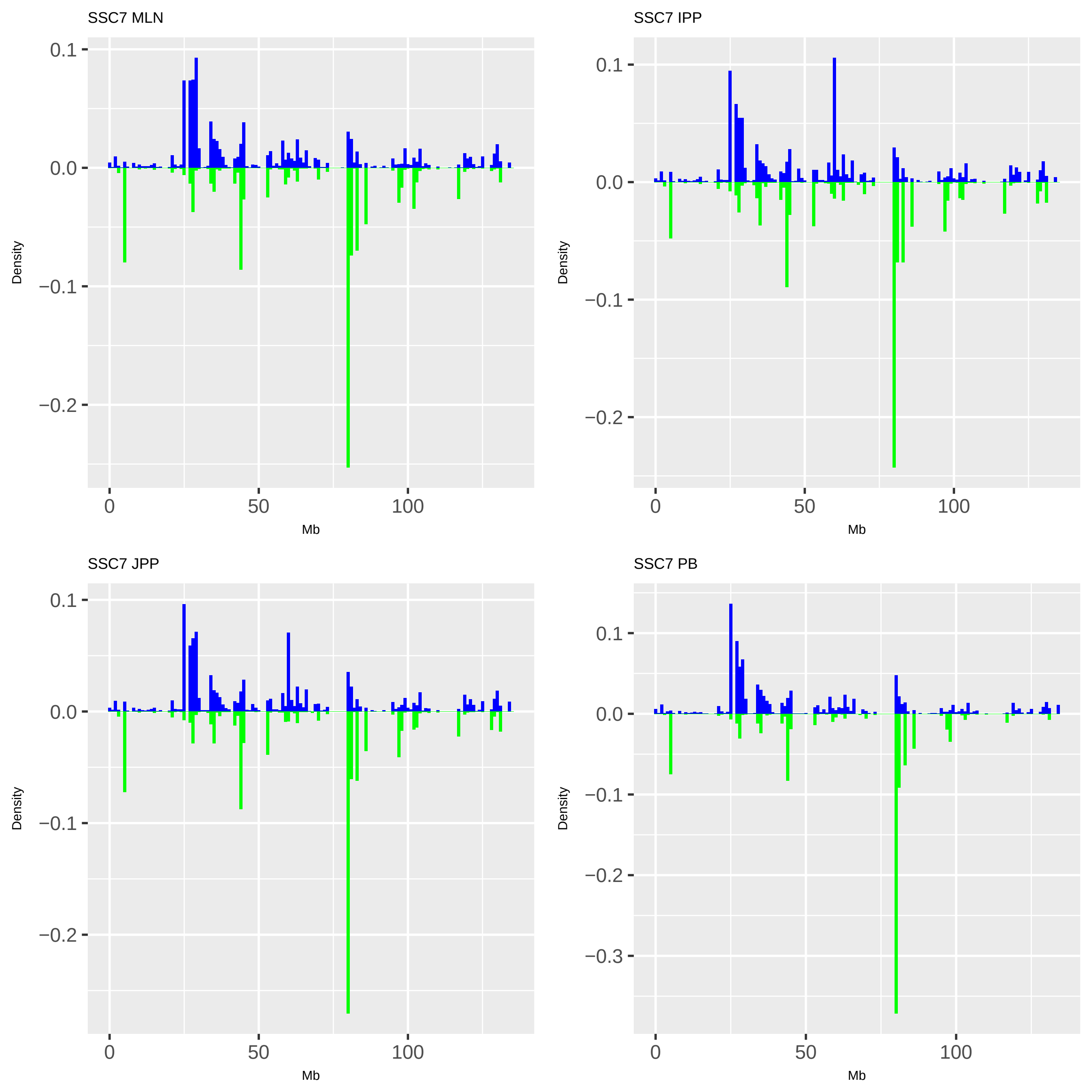

SSC7 MLN
SSC7 IPP
SSC7 JPP
SSC7 PB
Density
Mb
0.1
0.0
−0.1
−0.2
−0.3
0
50
100

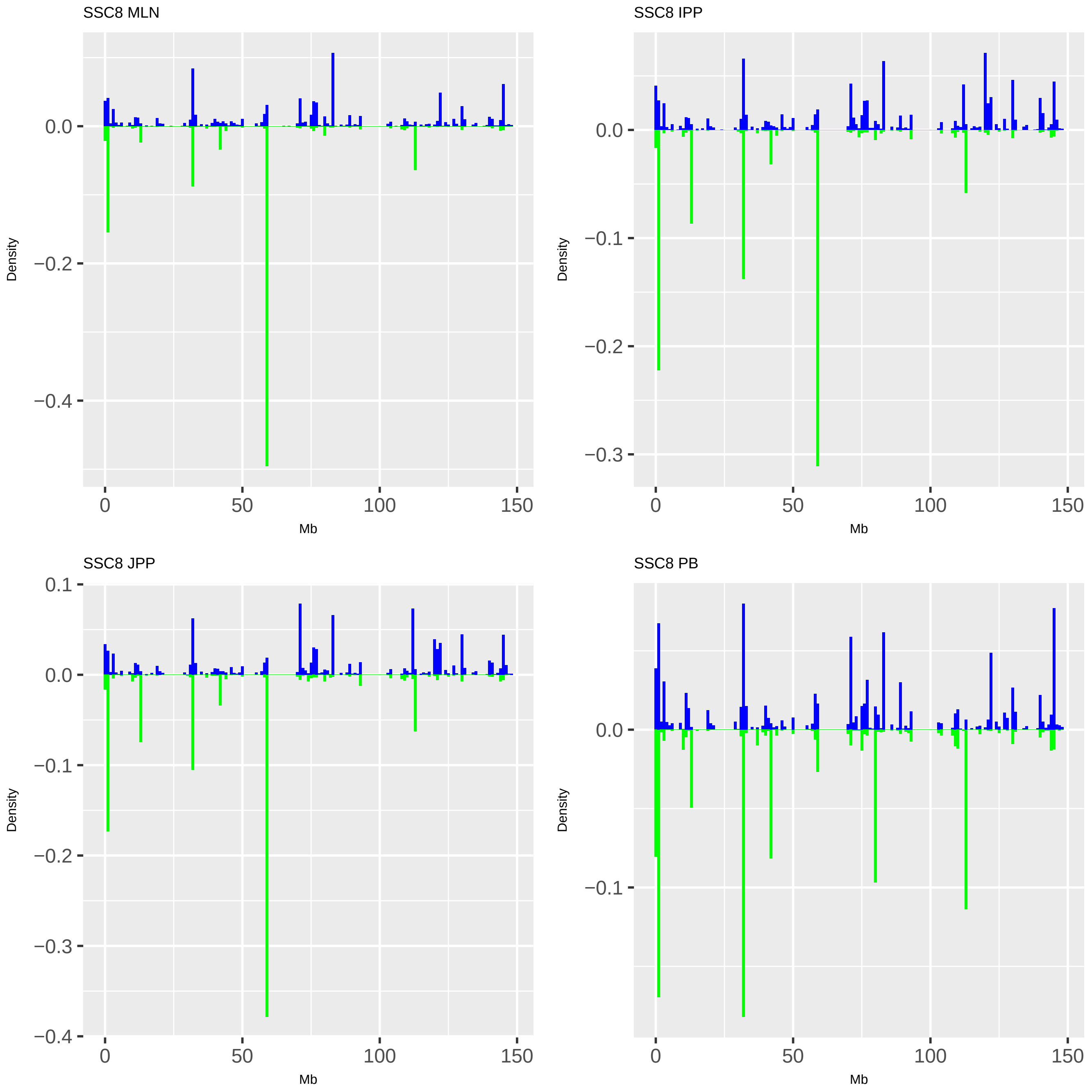
SSC8 MLN
SSC8 IPP
SSC8 JPP
SSC8 PB
Density
Mb
0.0
−0.2
−0.4
−0.1
−0.3
0.1
0
50
100
150

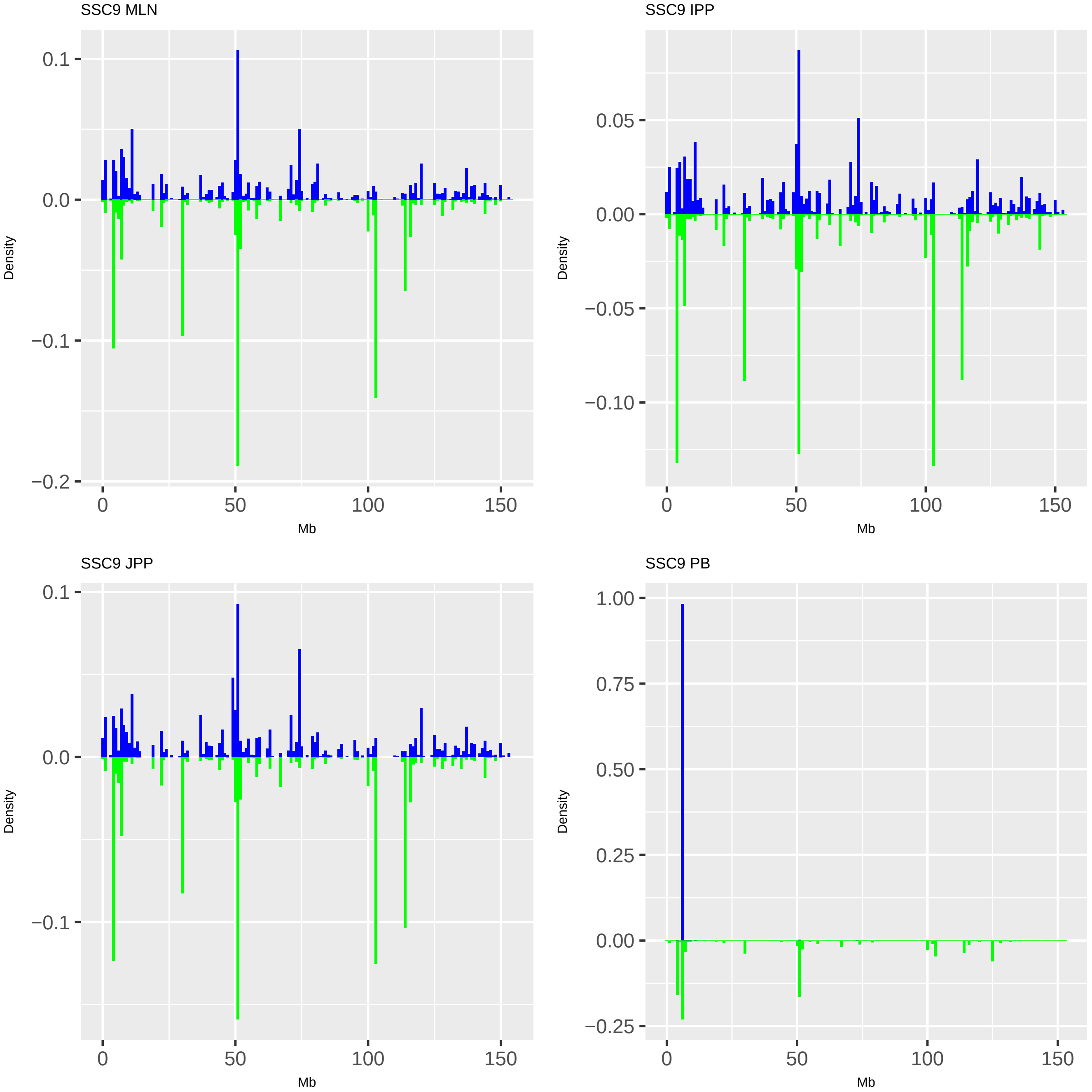

SSC9 MLN
SSC9 IPP
SSC9 JPP
SSC9 PB
Density
Mb
0
50
100
150
0.1
0.0
−0.1
−0.2
0.05
0.00
−0.05
−0.10
1.00
0.75
0.50
0.25
−0.25

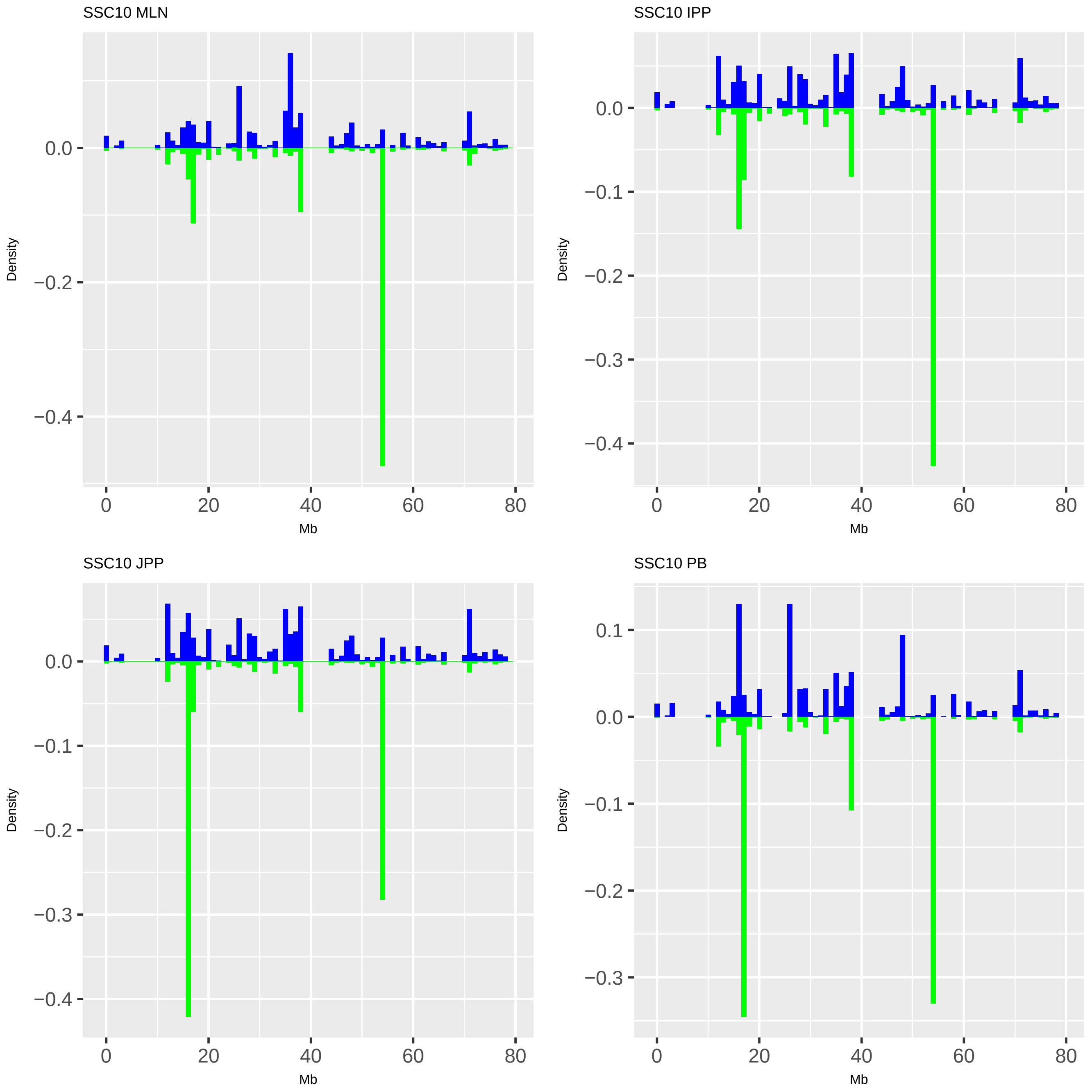

SSC10 MLN
SSC10 IPP
SSC10 JPP
SSC10 PB
Density
Mb
0.0
−0.1
−0.2
−0.3
−0.4
0
20
40
60
80

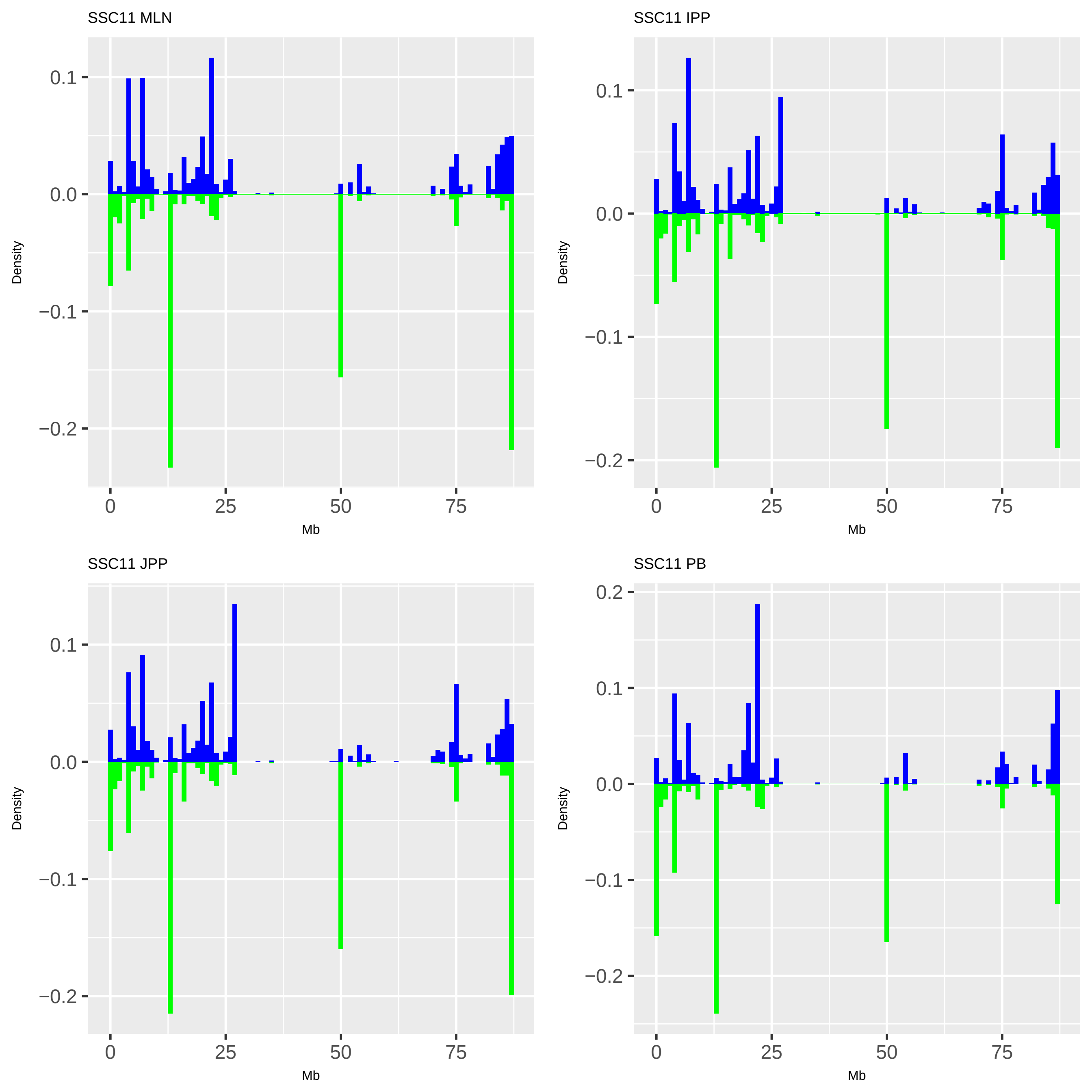
SSC11 MLN
SSC11 IPP
SSC11 JPP
SSC11 PB
Density
Mb
0.1
0.0
−0.1
−0.2
0
25
50
75

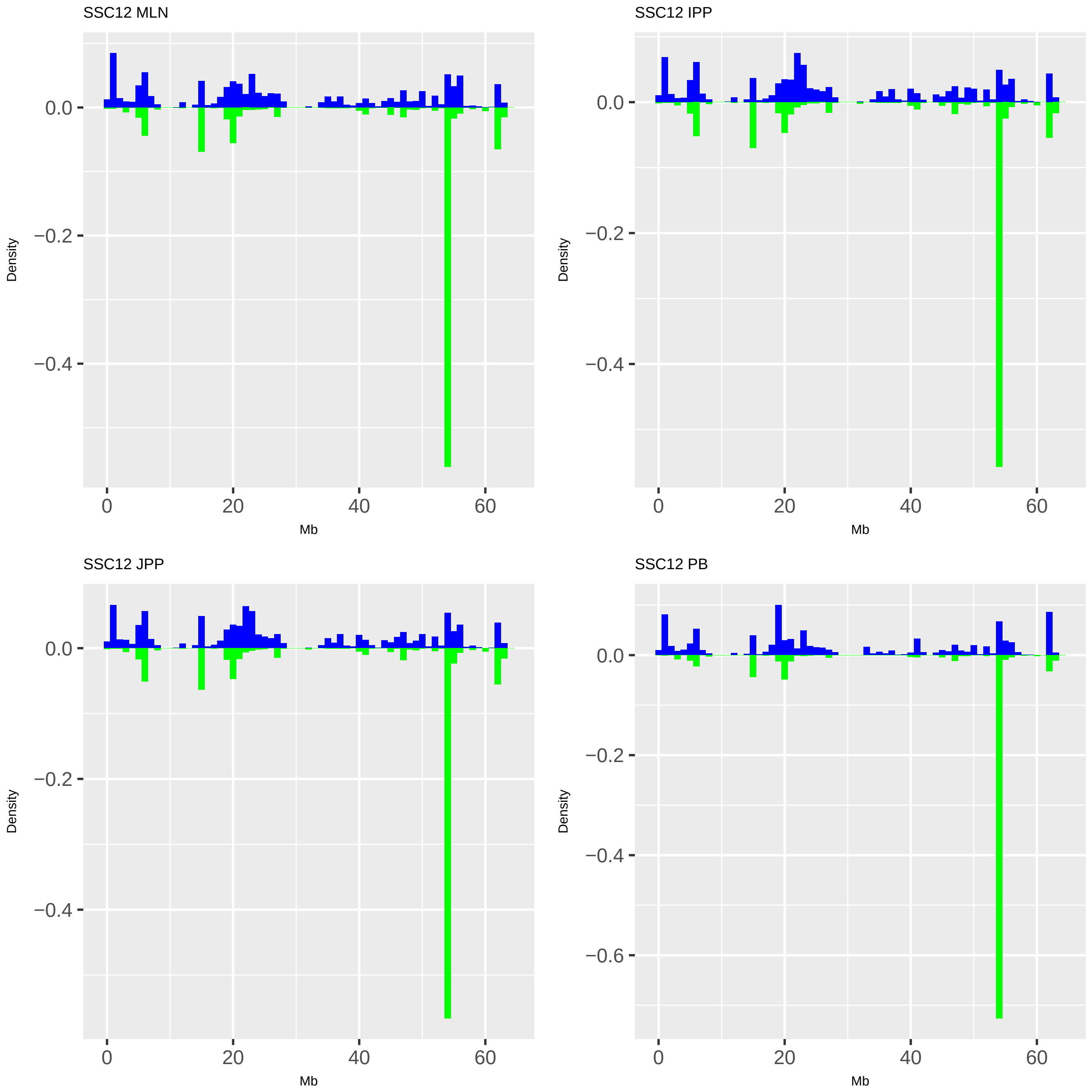
SSC12 MLN
SSC12 IPP
SSC12 JPP
SSC12 PB
Density
Mb
0.0
−0.2
−0.4
−0.6
0
20
40
60

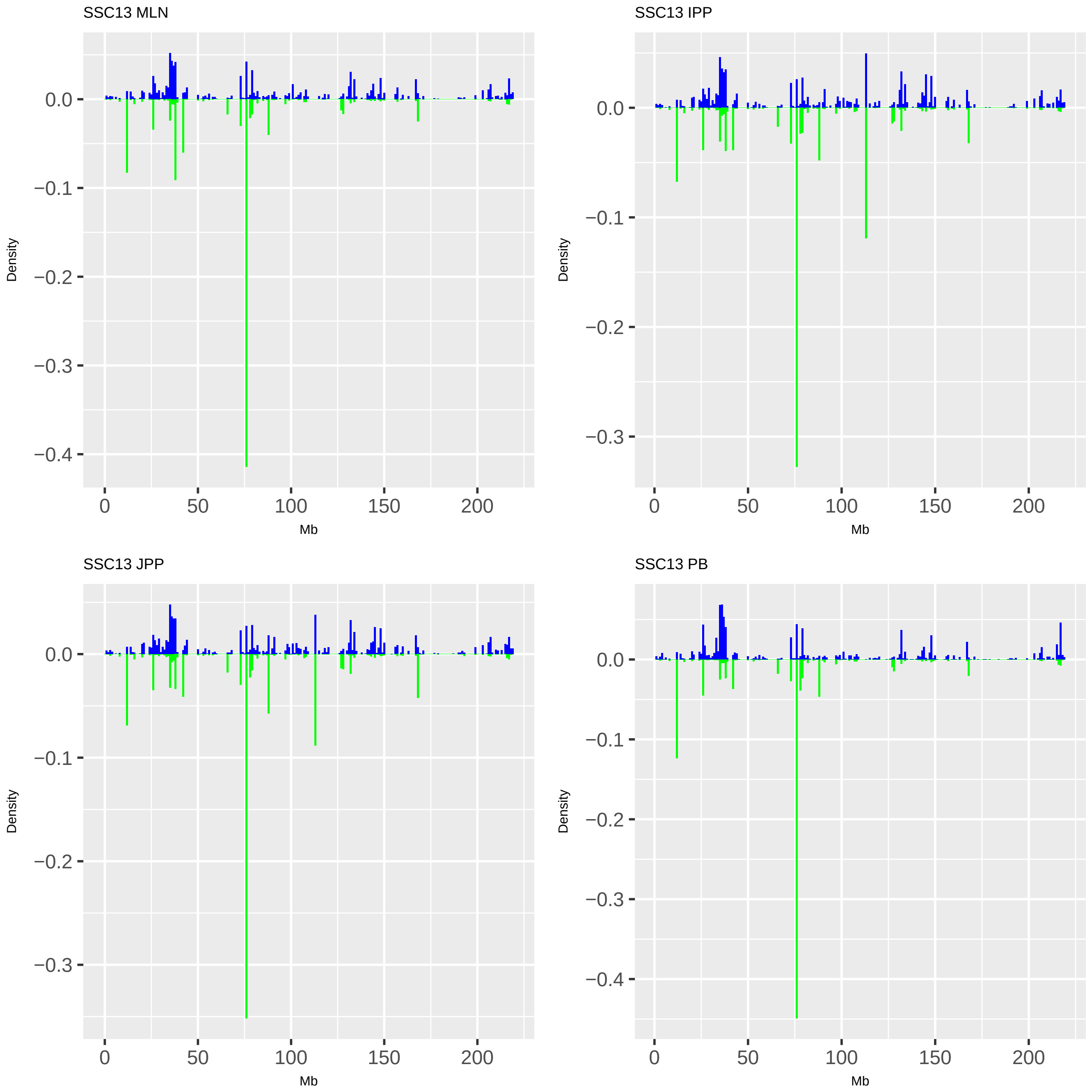
SSC13 MLN
SSC13 IPP
SSC13 JPP
SSC13 PB
Density
Mb
0.0
−0.1
−0.2
−0.3
−0.4
0
50
100
150
200

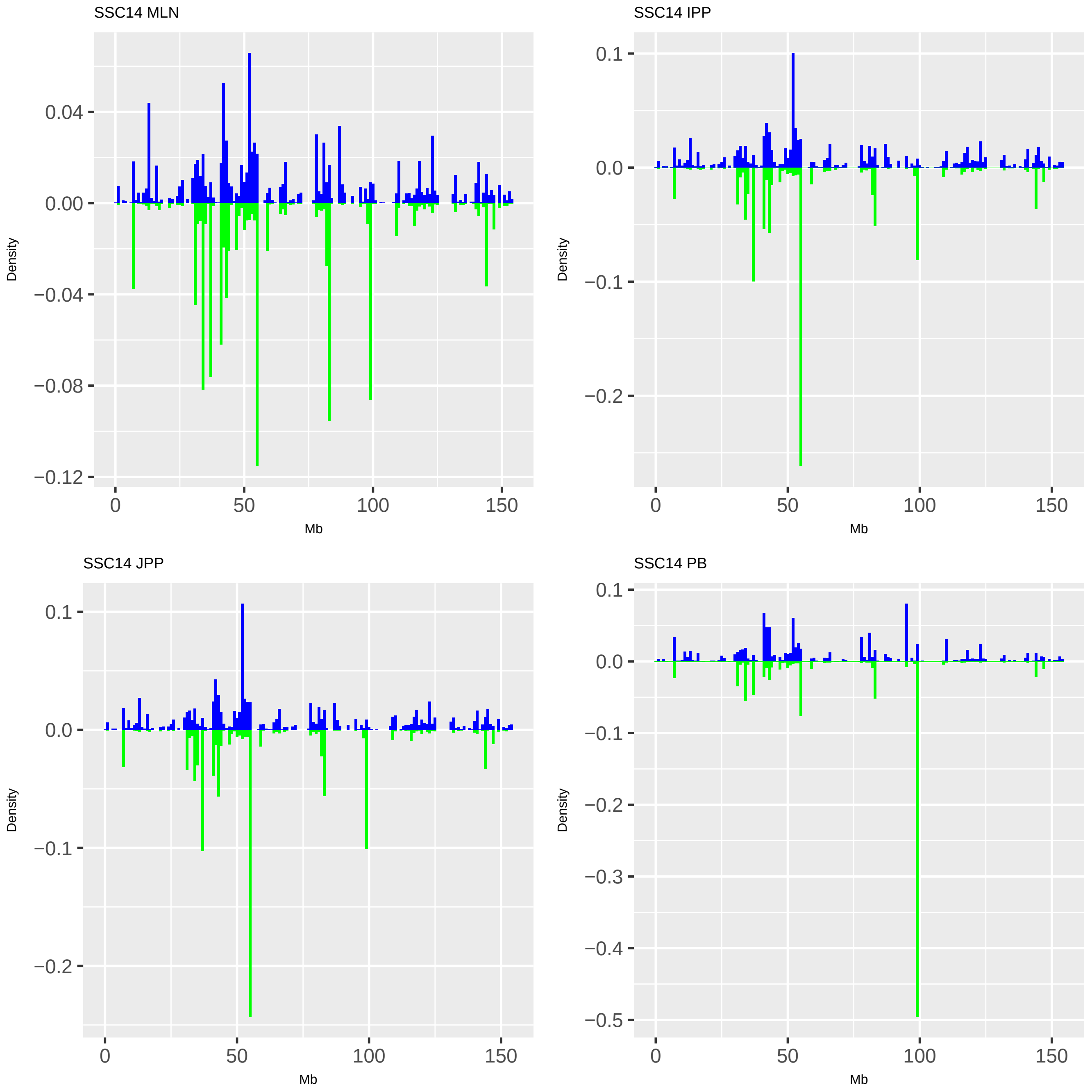

SSC14 MLN
0.04
0.00
−0.04
−0.08
−0.12
Density
0
50
100
150
Mb
SSC14 IPP
0.1
0.0
−0.1
−0.2
Density
0
50
100
150
Mb
SSC14 JPP
0.1
0.0
−0.1
−0.2
Density
0
50
100
150
Mb
SSC14 PB
0.1
0.0
−0.1
−0.2
−0.3
−0.4
−0.5
Density
0
50
100
150
Mb

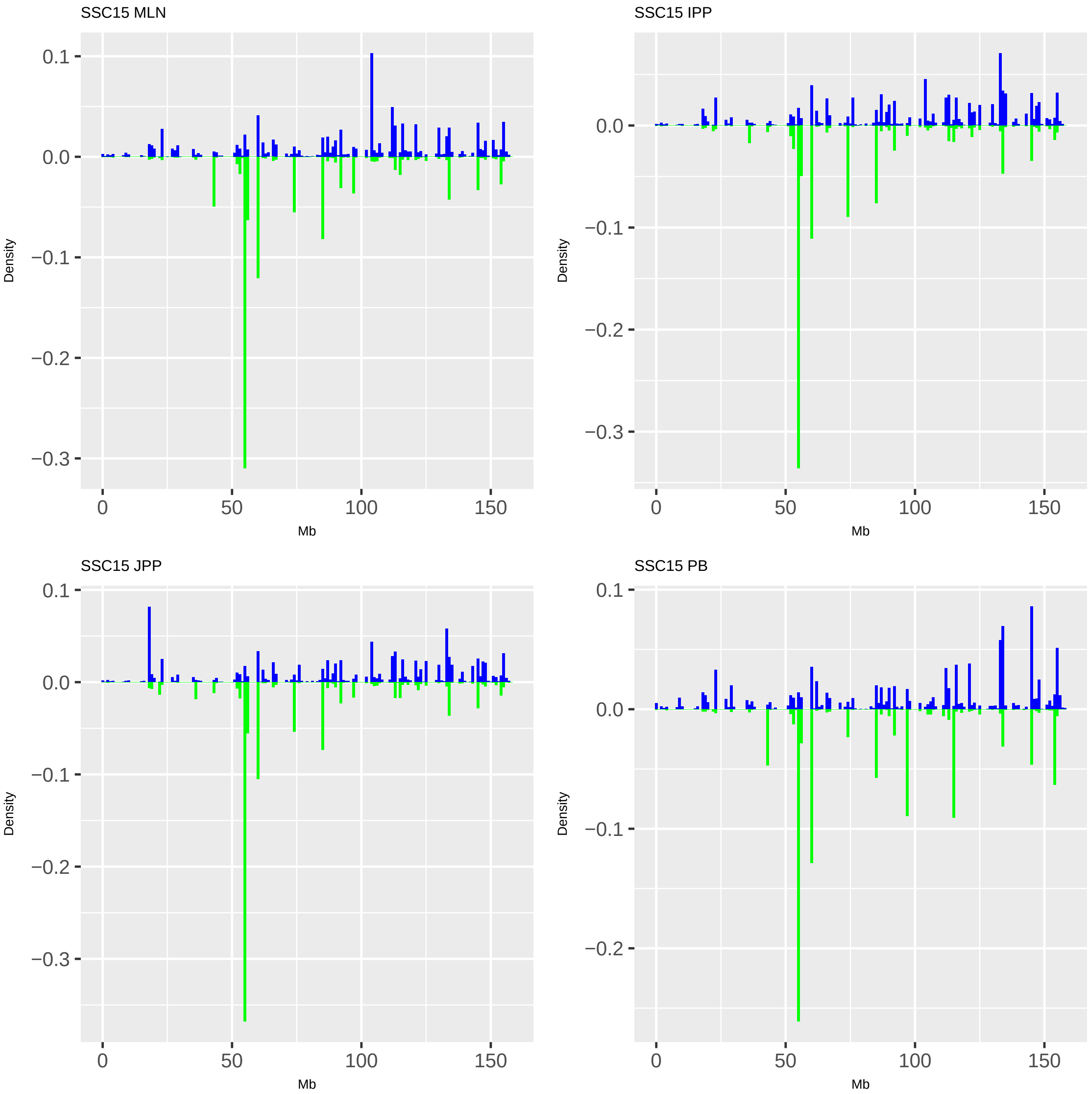
SSC15 MLN
0.1
0.0
−0.1
−0.2
−0.3
Density
0
50
100
150
Mb
SSC15 IPP
0.0
−0.1
−0.2
−0.3
Density
0
50
100
150
Mb
SSC15 JPP
0.1
0.0
−0.1
−0.2
−0.3
Density
0
50
100
150
Mb
SSC15 PB
0.1
0.0
−0.1
−0.2
Density
0
50
100
150
Mb

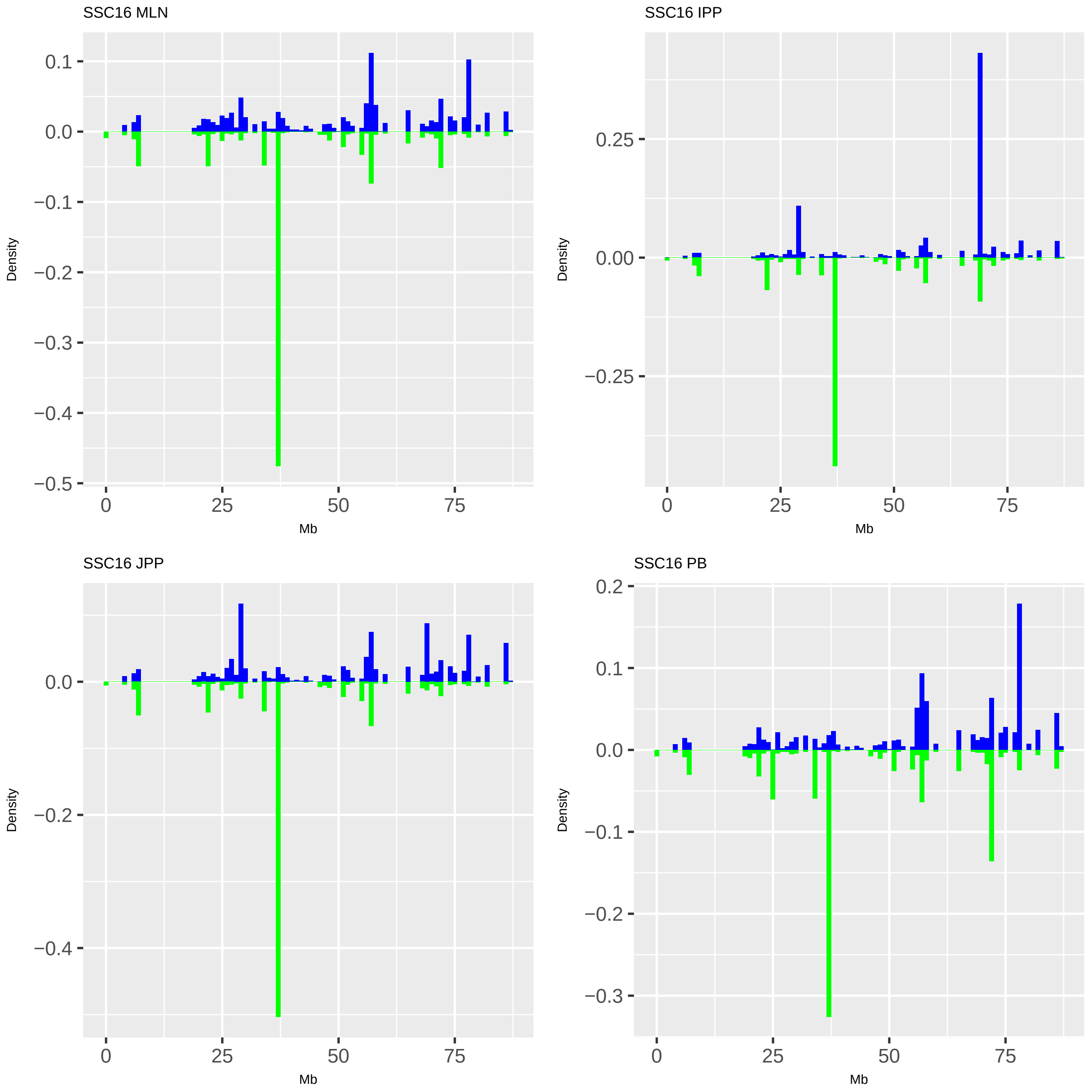
SSC16 MLN
SSC16 IPP
SSC16 JPP
SSC16 PB
Density
Mb
0.1
0.0
−0.1
−0.2
−0.3
−0.4
−0.5
0.25
0.00
−0.25
0.2
0
25
50
75

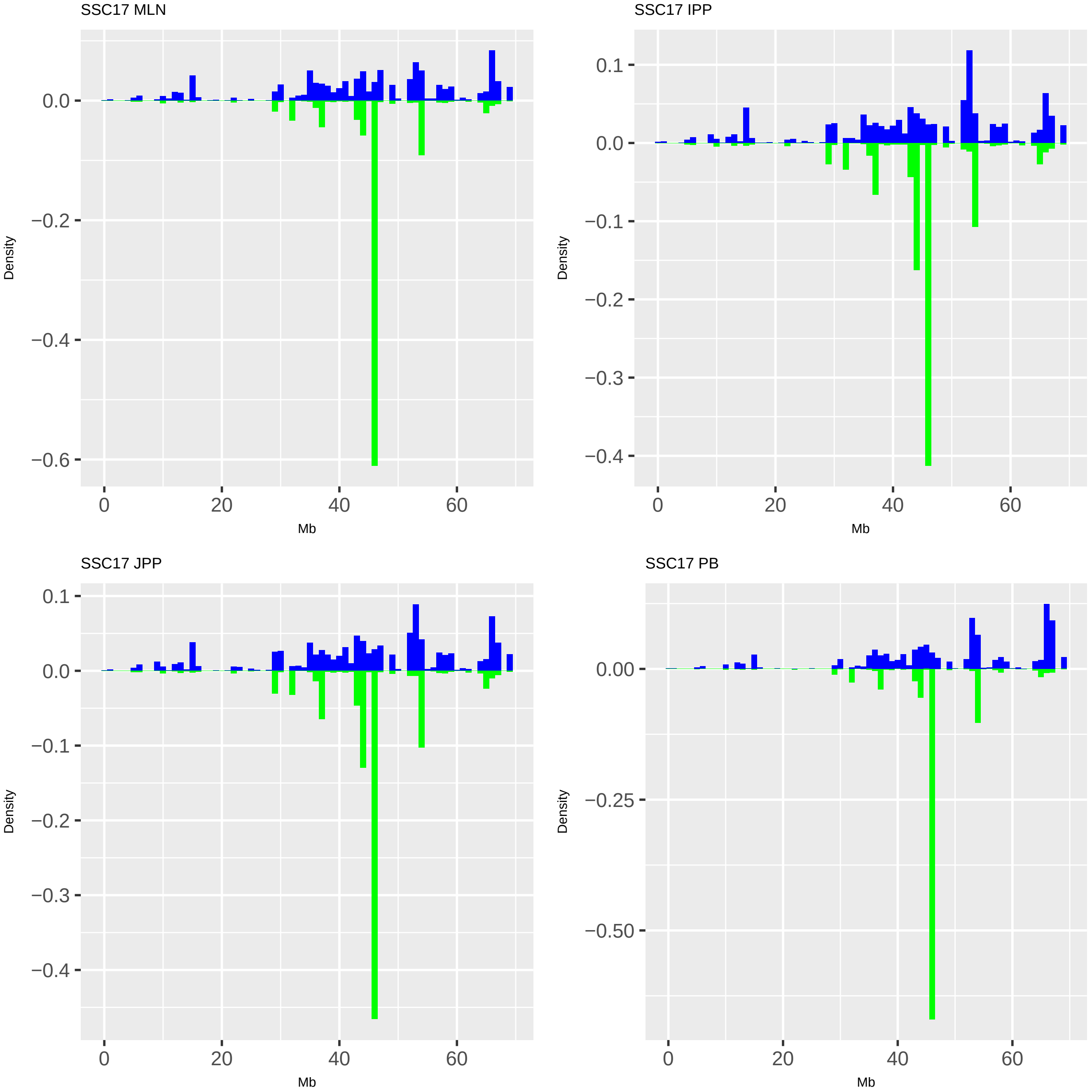

SSC17 MLN
0.0
−0.2
−0.4
−0.6
Density
0
20
40
60
Mb
SSC17 IPP
0.1
0.0
−0.1
−0.2
−0.3
−0.4
Density
0
20
40
60
Mb
SSC17 JPP
0.1
0.0
−0.1
−0.2
−0.3
−0.4
Density
0
20
40
60
Mb
SSC17 PB
0.00
−0.25
−0.50
Density
0
20
40
60
Mb

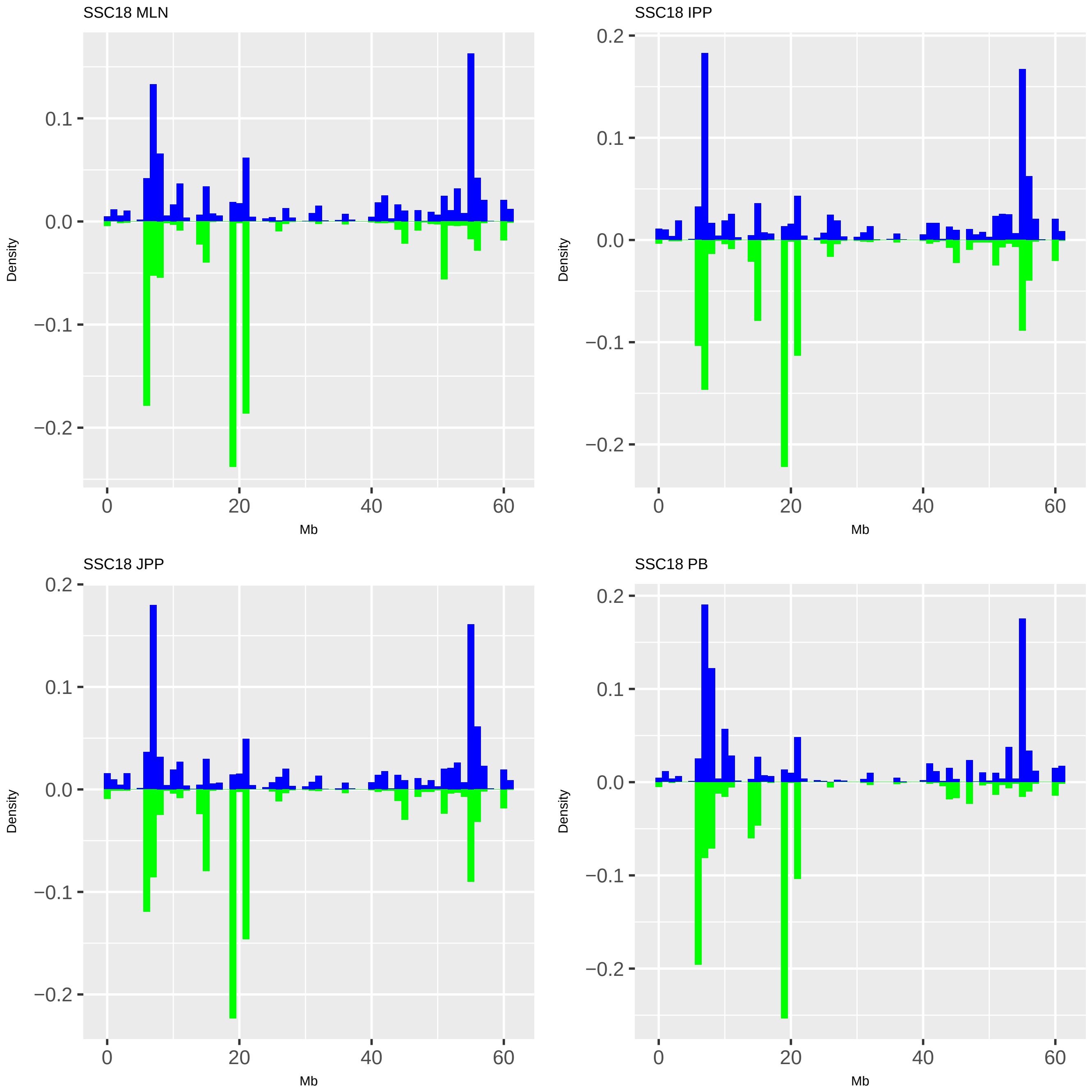
SSC18 MLN
SSC18 IPP
SSC18 JPP
SSC18 PB
Density
Mb
0.2
0.1
0.0
−0.1
−0.2
0
20
40
60
